# On the Choice of the Item Response Model for Scaling PISA Data: Model Selection Based on Information Criteria and Quantifying Model Uncertainty

**DOI:** 10.3390/e24060760

**Published:** 2022-05-27

**Authors:** Alexander Robitzsch

**Affiliations:** 1 IPN—Leibniz Institute for Science and Mathematics Education, Olshausenstraße 62, 24118 Kiel, Germany; robitzsch@leibniz-ipn.de; 2 Centre for International Student Assessment (ZIB), Olshausenstraße 62, 24118 Kiel, Germany

**Keywords:** item response model, scaling, PISA, model uncertainty

## Abstract

In educational large-scale assessment studies such as PISA, item response theory (IRT) models are used to summarize students’ performance on cognitive test items across countries. In this article, the impact of the choice of the IRT model on the distribution parameters of countries (i.e., mean, standard deviation, percentiles) is investigated. Eleven different IRT models are compared using information criteria. Moreover, model uncertainty is quantified by estimating model error, which can be compared with the sampling error associated with the sampling of students. The PISA 2009 dataset for the cognitive domains mathematics, reading, and science is used as an example of the choice of the IRT model. It turned out that the three-parameter logistic IRT model with residual heterogeneity and a three-parameter IRT model with a quadratic effect of the ability θ provided the best model fit. Furthermore, model uncertainty was relatively small compared to sampling error regarding country means in most cases but was substantial for country standard deviations and percentiles. Consequently, it can be argued that model error should be included in the statistical inference of educational large-scale assessment studies.

## 1. Introduction

Item response theory (IRT) models [1] are central to analyzing dichotomous random variables. IRT models can be regarded as a factor-analytic multivariate technique to summarize a high-dimensional contingency table by a few latent factor variables of interest. Of particular interest is the application of an IRT model in educational large-scale assessment (LSA; [2]), such as the programme for international student assessment (PISA; [3]), which summarizes the ability of students on test items in different cognitive domains.

In the official reporting of outcomes of LSA studies such as PISA, the set of test items is represented by a unidimensional summary measure extracted by applying a unidimensional IRT model. Across different LSA studies, there is no consensus on which particular IRT model should be utilized [4,5,6]. In previous research, there are a few attempts that quantity the impact of IRT model choice on distribution parameters of interest such as country means, standard deviations, or percentiles. However, previous research did not systematically study a large number of competing IRT models [7,8,9]. Our research fills a gap because it conducts an empirical comparison involving 11 different IRT models for scaling for PISA 2009 data in three ability domains. Moreover, we compare the model fit of these different IRT models and quantify the variability in model uncertainty using the model error. We compare the model error with the standard error associated with the uncertainty due to the sampling of students.

The rest of the article is structured as follows. In Section 2, we discuss different IRT models used for scaling. Section 3 introduces the concepts of model selection and model uncertainty. Section 4 describes the method used to analyze PISA 2009 data. In Section 5, we discuss the empirical results for the PISA 2009 dataset. Finally, the paper closes with a discussion in Section 6.

## 2. Item Response Models for Scaling Cognitive Test Items

In this section, we present an overview of different IRT models that are used for scaling cognitive test data to obtain a unidimensional summary score [10,11,12]. In the rest of the article, we restrict ourselves to the treatment of dichotomous items. However, the principle can similarly be applied to polytomous items.

Let $X=(X_{1},\ldots ,X_{I})$ be the vector of *I* dichotomous items $X_{i}\in \left\{0,1\right\}$. A unidimensional IRT model [11,12] is a statistical model for the probability distribution P(X=x) for $x\in {\left\{0,1\right\}}^{I}$, where

(1) $$P\left(X=x;\gamma \right)=\int_{-\infty }^{\infty }\prod_{i=1}^{I}\left[{P_{i}\left(\theta ;\gamma_{i}\right)}^{x_{i}}{\left(1-P_{i}\left(\theta ;\gamma_{i}\right)\right)}^{1-x_{i}}\right]f\left(\theta \right)\;d\theta ,\;\theta ∼F.$$

In Equation (1), a latent variable θ is involved that can be interpreted as a unidimensional summary of the test items X. The distribution of θ is modeled using a (semi)parametric distribution *F* with density function *f*. In the rest of the article, we fix this distribution to be standard normal, but this can be weakened [13,14,15]. The item response functions (IRF) $P_{i}\left(\theta ;\gamma_{i}\right)$ model the relationship of the dichotomous item with the latent variable, and we collect all item parameters in the vector γ. In most cases, a parametric model is utilized in the estimation of the IRF (but see [16] for a nonparametric identification), which is indicated by the item parameter $\gamma_{i}$ in Equation (1). Note that in (1), item responses $X_{i}$ are conditionally independent on θ; that is, after controlling the latent ability θ, pairs of items $X_{i}$ and $X_{j}$ are conditionally uncorrelated. This property is also known as the local dependence assumption, which can be statistically tested [12,17]. The item parameters $\gamma_{i}$ of the estimated IRFs in Equation (1) can be estimated by (marginal) maximum likelihood (ML) using an EM algorithm [18,19,20]. The estimation can involve sampling weights for students [21] and a multi-matrix design in which only a subset of items is administered to each student [22]. In the likelihood formulation of (1), non-administered items are skipped in the multiplication term.

In practice, the IRT model (1) is likely to be misspecified because the unidimensionality assumption is implausible. Moreover, the parametric assumption $P_{i}\left(\theta ;\gamma_{i}\right)$ of the IRF might be incorrect. In addition, in educational LSA studies involving a large number of countries, there will typically be country differential item functioning [23,24,25]; that is, item parameters will vary across countries. In this case, applying ML using country-invariant item parameters defines the best approximation with respect to the Kullback–Leibler distance of the true distribution and a model-implied distribution. In this sense, an IRT model is selected by purpose and not by reasons of model fit because it will not even approximately fit the data (see also [26]). If country means are computed based on a particular IRT model, the parameter of interest should be, rather, interpreted as a descriptive statistic of interest [27]. Using a particular model does not mean that we believe that the model (approximately) fits the data. In contrast, we think that a vector of country means μ and item parameters γ summarize a high-dimensional contingency table P(X=x).

Locally optimal weights [28] can be used to discuss the consequences for scoring when using a particular IRT model. A local scoring rule for the ability θ can be defined by a weighted sum $\sum_{i=1}^{I}\nu_{i}\left(\theta \right)X_{i}$ for abilities near $\theta =\theta_{0}$. The ability θ is determined by ML estimation using previously estimated item parameters. The locally optimal weights can be derived as (see [27,28,29]):

(2) $$\nu_{i}\left(\theta \right)=\frac{P_{i}^{\prime }\left(\theta \right)}{P_{i}\left(\theta \right)\left(1-P_{i}\left(\theta \right)\right)}$$

If the local weight $\nu_{i}\left(\theta \right)$ (also referred to as the local item score) varies across different θ values, the impact of single items in the ability differs. This property can be critically recognized, particularly for country comparisons in LSA studies [29]. Subsequently, we will discuss the properties of different IRT models regarding the optimal weights $\nu_{i}\left(\theta \right)$.

In this article, several competitive functional forms of the IRF are compared, and their consequences for distribution parameters (e.g., means, standard deviations, and percentiles) for the prominent LSA study PISA are discussed. Performing such a fit index contest [30,31] does not necessarily mean that we favor model selection based on model fit. In the next Section 2.1, we discuss several IRFs later utilized for model comparisons. In Section 2.2, we investigate the behavior of the estimated ability distribution under misspecified IRFs. Finally, we conclude this section with some thoughts on the choice of the IRT model (see Section 2.3).

### 2.1. Different Functional Forms for IRT Models

In this section, we discuss several parametric specifications of the IRF $P_{i}\left(\theta \right)$ that appear in the unidimensional IRT model defined in Equation (1).

The one-parameter logistic model (1PL; also known as the Rasch model; [32,33]) employs a logistic link function and parametrizes an item with a single parameter $b_{i}$ that is called item difficulty. The model is defined by

(3) $$\;\;\mathrm{Model}\;1\mathrm{PL}\;:\;P_{i}\left(\theta \right)=\frac{1}{1+\exp(-a\;\theta -b_{i})}\;,$$

where *a* is the common item discrimination parameter. Alternatively, one can fix the parameter *a* to 1 and estimate the standard deviation of the latent variable θ. Notably, the sum score $\sum_{i=1}^{I}x_{i}$ is a sufficient statistic for θ in the 1PL model. The 1PL model has wide applicability in educational assessment [34,35].

The 1PL model uses a symmetric link function. However, asymmetric link functions could also be used for choosing an IRF. The cloglog link function is used in the one-parameter cloglog (1PCL) model [36,37]:

(4) $$\;\;\mathrm{Model}\;1\mathrm{PCL}\;:\;P_{i}\left(\theta \right)=1-\exp\left(-\exp\left(a\;\theta +b_{i}\right)\right)\;.$$

Consequently, items are differentially weighted in the estimation of θ at each θ location, and the sum score is not a sufficient statistic. The cloglog link function has similar behavior to the logistic link function in the 1PL model in the lower tail (i.e., for negative values of θ), but differs from it in the upper tail.

The one-parameter loglog (1PLL) IRT model is defined by

(5) $$\;\;\mathrm{Model}\;1\mathrm{PLL}\;:\;P_{i}\left(\theta \right)=\exp\left(-\exp\left(-a\;\theta -b_{i}\right)\right)\;.$$

In contrast to the cloglog link function, the loglog function is similar to the logistic link function in the upper tail (i.e., for positive θ values), but different from it in the lower tail.

**Figure 1:**
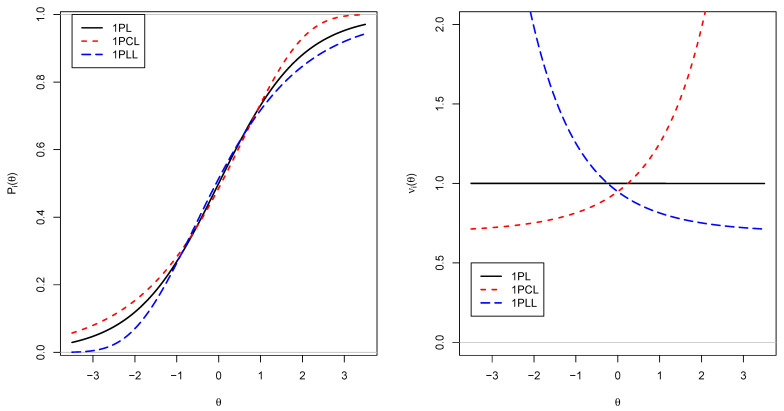
Item response functions $P_{i}$ (**left panel**) and locally optimal weights $\nu_{i}$ (**right panel**) for the 1PL, 1PCL and 1PLL models.

Figure 1 compares the 1PL, 1PCL, and 1PLL models regarding the IRF $P_{i}$ and the locally optimal weight $\nu_{i}$. The loglog IRT model (1PLL) stretches more in the lower tails than in the lower θ tail than the logistic link function. The converse is true for the cloglog IRT model (1PCL), which is significantly stretched in the upper θ tail. In the right panel of Figure 1, locally optimal weights are displayed. The 1PL model has a constant weight of 1, while the local contribution of item score for θ differs across the θ range for the 1PCL and the 1PLL model. The 1PCL model provides a higher local item score for higher θ values than for lower θ values. Hence, more difficult items receive lower local item scores than easier items. In contrast, the 1PLL model results in higher local item scores for difficult items compared to easier items. This idea is reflected in the D-scoring method [38,39].

Notably, the 1PCL and 1PLL models use asymmetric IRFs. One can try to estimate the extent of asymmetry in IRFs by using a generalized logistic link function (also called the Stukel link function; [40]):

(6) $$\;\;\mathrm{Model}\;1\mathrm{PGL}\;:\;P_{i}\left(\theta \right)=\frac{1}{1+\exp(-S\left(a\;\theta +b_{i};\alpha_{1},\alpha_{2}\right))}\;,$$

where the generalized logit link function is defined as

(7) $$S\left(x;\alpha_{1},\alpha_{2}\right)=\left\{\begin{array}{cc} \alpha_{1}^{-1}\left(\exp(\alpha_{1}x)-1\right) & \mathrm{if}\;x\geq 0\;\mathrm{and}\;\alpha_{1}>0\\ x & \mathrm{if}\;x\geq 0\;\mathrm{and}\;\alpha_{1}=0\\ -\alpha_{1}^{-1}\log\left(1-\alpha_{1}x\right) & \mathrm{if}\;x\geq 0\;\mathrm{and}\;\alpha_{1}<0\\ -\alpha_{2}^{-1}\left(\exp(-\alpha_{2}x)-1\right) & \mathrm{if}\;x<0\;\mathrm{and}\;\alpha_{2}>0\\ x & \mathrm{if}\;x<0\;\mathrm{and}\;\alpha_{2}=0\\ \alpha_{2}^{-1}\log\left(1+\alpha_{2}x\right) & \mathrm{if}\;x<0\;\mathrm{and}\;\alpha_{2}<0 \end{array}\right.$$

In this 1PGL model, common shape parameters $\alpha_{1}$ and $\alpha_{2}$ for the IRFs are additionally estimated. The 1PL, 1PCL and 1PLL models can be obtained as special cases of (6).

The four models 1PL, 1PCL, 1PLL, and 1PGL have in common that they only estimate one parameter per item. The assumption of a common item discrimination is weakened in the two-parameter logistic (2PL) IRT model [28], as a generalization of the 1PL model in which the discriminations $a_{i}$ are now made item-specific:

(8) $$\;\;\mathrm{Model}\;2\mathrm{PL}\;:\;P_{i}\left(\theta \right)=\frac{1}{1+\exp(-a_{i}\theta -b_{i})}\;.$$

Note that $\sum_{i=1}^{I}a_{i}x_{i}$ is a sufficient statistic for θ. Hence, items $X_{i}$ are differentially weighted by the weight $a_{i}$, which is determined within the statistical model.

Further, the assumption of a symmetric logistic link function might be weakened, and a four-parameter generalized logistic (4PGL) model can be estimated:

(9) $$\;\;\mathrm{Model}\;4\mathrm{PGL}\;:\;P_{i}\left(\theta \right)=P_{i}\left(\theta \right)=\frac{1}{1+\exp(-S\left(a\;\theta +b_{i};\alpha_{1i},\alpha_{2i}\right))}\;.$$

In the IRT model (9), the shape parameters $\alpha_{1i}$ and $\alpha_{2i}$ are made item-specific. Hence, the extent of asymmetry of the IRF is estimated for each item.

The 2PL model (8) can be generalized to the three-parameter logistic (3PL; [41]) IRT model that assumes an item-specific lower asymptote $c_{i}$ larger than 0 for the IRF:

(10) $$\;\;\mathrm{Model}\;3\mathrm{PL}\;:\;P_{i}\left(\theta \right)=c_{i}+\left(1-c_{i}\right)\frac{1}{1+\exp(-a_{i}\theta -b_{i})}\;.$$

Parameter $c_{i}$ is often referred to as a (pseudo-)guessing parameter [42,43]. The 3PL model might be reasonable if multiple-choice items are used in the test.

The 3PL model can be generalized in the four-parameter logistic (4PL; [44,45,46]) model such that it also contains upper asymptotes $d_{i}$ smaller than 1 for the IRF:

(11) $$\;\;\mathrm{Model}\;4\mathrm{PL}\;:\;P_{i}\left(\theta \right)=c_{i}+\left(1-d_{i}-c_{i}\right)\frac{1}{1+\exp(-a_{i}\theta -b_{i})}\;.$$

The $d_{i}$ parameter is often referred to as a slipping parameter, which characterizes careless (incorrect) item responses [47]. In contrast to the 1PL, 2PL, or the 3PL model, the 4PL model has not yet been applied in the operational practice of LSA studies. However, there are a few research papers that apply the 4PL model to LSA data [48,49].

It should be mentioned that the 3PL or the 4PL model might suffer from empirical nonidentifiability [45,50,51,52]. This is why prior distributions for guessing (3PL and 4PL) and slipping (4PL) parameters are required for stabilizing model estimation. As pointed out by an anonymous reviewer, the use of prior distributions changes the meaning of the IRT model. However, we think that identifiability issues are of less concern in the large-sample-size situations that are present in educational LSA studies. If item parameters are obtained in a pooled sample of students comprising all countries, sample sizes are typically above 10,000. In this case, the empirical data will typically dominate prior distributions, and prior distributions are therefore not needed.

**Figure 2:**
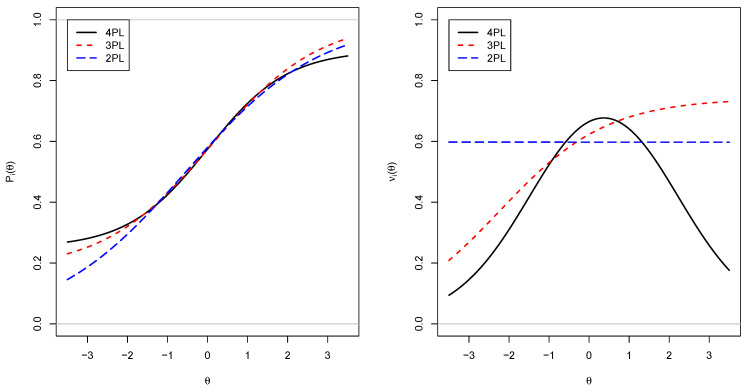
Item response functions $P_{i}$ (**left panel**) and locally optimal weights $\nu_{i}$ (**right panel**) for the 4PL, 3PL and 2PL models.

In Figure 2, IRFs and locally optimal weights for the 4PL, 3PL, and 2PL models are displayed. The item parameters for the 4PL model were $a_{i}=1$, $b_{i}=0$, $c_{i}=0.25$, and $d_{i}=0.1$. The parameters of the displayed 2PL and 3PL models were obtained by minimizing the weighted squared distance between the IRF of the 4PL model and the simpler model under the constraint that the model-implied item-means coincide under the normal distribution assumption of θ. Importantly, it can be seen in the right panel that the 2PL model has a constant local item score, while it is increasing for the 3PL model and it is inversely U-shaped for the 4PL model. Hence, when using the 4PL model, it must not be too easy or too difficult to obtain a high local item score for a student that got the item correct.

A different strand of model extensions also starts from the 2PL model but introduces more item parameters to model asymmetry or nonlinearity while retaining the logistic link function. The three-parameter logistic model with quadratic effects (3PLQ) additionally includes additional quadratic effects of θ in the 2PL model [42,50]:

(12) $$\;\;\mathrm{Model}\;3\mathrm{PLQ}\;:\;P_{i}\left(\theta \right)=\frac{1}{1+\exp(-a_{2i}\theta^{2}-a_{1i}\theta -b_{i})}\;.$$

Due to the presence of the $a_{2i}$ parameter, asymmetric IRFs can be modeled. As a disadvantage, the IRF in (12) must not be monotone, although this constraint can be incorporated in the estimation [53,54].

The three-parameter model with residual heterogeneity (3PLRH) extends to the 2PL model by including an asymmetry parameter $\delta_{i}$ [55,56]:

(13) $$\;\;\mathrm{Model}\;3\mathrm{PLRH}\;:\;P_{i}\left(\theta \right)=\frac{1}{1+\exp\left(-{\left\{1+\exp(-\delta_{i}\theta )\right\}}^{1/2}\left(a_{i}\theta +b_{i}\right)\right)}\;.$$

The 3PLRH model has been successfully applied to LSA data and often resulted in superior model fit compared to the 3PL model [57,58].

**Figure 3:**
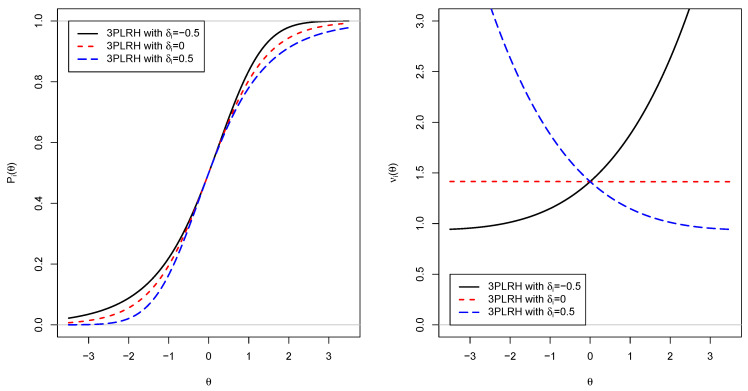
Item response functions $P_{i}$ (**left panel**) and locally optimal weights $\nu_{i}$ (**right panel**) for different IRFs of the 3PLRH model.

In Figure 3, IRFs and locally optimal weights are displayed for three parameter specifications in the 3PLRH model (i.e., $a_{i}=1$, $b_{i}=0$, and $\delta_{i}=-0.5,0,0.5$). One can see that the introduced asymmetry parameter $\delta_{i}$ governs the behavior of the IRF in the lower or upper tails. The displayed IRFs mimic the 1PL, 1PCL, and 1PLL models. Moreover, with $\delta_{i}$ parameters different from zero, different locally optimal weights across the θ range are introduced. Notably, a positive $\delta_{i}$ parameter is associated with a larger local item score in the lower θ tail. The opposite is true for a negative $\delta_{i}$ parameter.

Finally, the 3PL model is extended in the four-parameter logistic model with quadratic effects (4PLQ), in which additional item-specific quadratic effects for θ are included [50]

(14) $$\;\;\mathrm{Model}\;4\mathrm{PLQ}\;:\;P_{i}\left(\theta \right)=c_{i}+\left(1-c_{i}\right)\frac{1}{1+\exp(-a_{i2}\theta^{2}-a_{i1}\theta -b_{i})}\;.$$

### 2.2. Ability Estimation under Model Misspecification

In this section, we study the estimation of θ when working with a misspecified IRT model. In the treatment, we assume that there is a true IRT model with unknown IRFs. We study the bias in estimated abilities for a fixed value of θ if misspecified IRFs are utilized. This situation refers to the empirical application in an LSA study, in which a misspecified IRF is estimated based on data comprising all countries, and the distribution of θ is evaluated at the level of countries. The misspecification emerges due to incorrectly assumed functional forms of the IRF or the presence of differential item functioning at the level of countries [24,59].

We assume that the there are true but unknown IRFs $P_{i}\left(\theta \right)=\Psi \left(\alpha_{i}\left(\theta \right)\right)$ with a continuously differentiable function $\alpha_{i}$ and $\Psi \left(x\right)={\left[1+\exp\left(-x\right)\right]}^{-1}$ denotes the logistic link function. We assume that the local independence assumption holds in the IRT model. For estimation, we use a misspecified IRT model with IRFs $P_{i}\left(\theta \right)=\Psi \left(a{\;}_{i}\left(\theta \right)\right)$ with a continuously differentiable function $a_{i}$. Notably, there exists a misspecification if $\alpha_{i}\neq a_{i}$. In Appendix A, we derive an estimate $\theta_{1}$ under the misspecified IRT model if $\theta_{0}$ is the data-generating ability value under the true IRT model. Hence, we derive a transformation function $m\left(\theta_{1}\right)=\theta_{0}+B\left(\theta_{0}\right)$, where B(θ) is the bias function that indicates the bias in the estimated ability due to the application of the misspecified IRT model. We assume that the item parameters under the misspecified IRT model are known (i.e., the IRFs $a_{i}\left(\theta \right)$ are known). Then, the ML estimate is determined based on the misspecified IRT model taking into account that $\theta_{0}$ solves the maximum likelihood equation under the true IRT model. It is assumed that the number of items *I* is large. Moreover, we apply two Taylor approximations that rely on the assumption that $|\alpha_{i}\left(\theta \right)-a_{i}\left(\theta \right)|$ is sufficiently small.

The derivation in Appendix A (see Equation (A10)) provides

(15) $$\theta_{1}≃\theta_{0}+A^{-1}\sum_{i=1}^{I}\left[\Psi \left(a_{i}\left(\theta_{0}\right)\right)-\Psi \left(\alpha_{i}\left(\theta_{0}\right)\right)\right]\alpha_{i}^{\prime }\left(\theta_{0}\right)\equiv \theta_{0}+B\left(\theta_{0}\right)\;,$$

where the bias term *B* is defined by $B\left(\theta \right)=A^{-1}\sum_{i=1}^{I}\left[\Psi \left(a_{i}\left(\theta \right)\right)-\Psi \left(\alpha_{i}\left(\theta \right)\right)\right]\alpha_{i}^{\prime }\left(\theta \right)$ and *A* is determined by item information functions (see Appendix A). Equation (15) clarifies how the misspecified IRFs enter the computation of θ. Interestingly, the extent of misspecification $\Psi \left(a_{i}\left(\theta_{0}\right)\right)-\Psi (\alpha_{i}\left(\theta_{0}\right)$ is weighted by $\alpha_{i}^{\prime }\left(\theta_{0}\right)$.

Equation (15) provides practical consequences when applying misspecified IRT models. For instance, $\theta_{0}$ might be the true country percentile, referring to a true IRT model. If the transformation $\theta_{1}=m\left(\theta_{0}\right)$ is monotone, the percentile with the misspecified model is $\theta_{1}$ and Equation (15) quantifies a bias for the estimated percentile. Moreover, let $f_{c}$ be the density of the ability under the true IRT model for country *c*; then, one can determine the bias in the country means by using (15). The true country mean of country *c* is given by $\mu_{c}=\int \theta f_{c}\left(\theta \right)\;d\theta$. The estimated country mean $\mu_{c}^{*}$ under the misspecified model is given by

(16) $$\mu_{c}^{*}=\mu_{c}+\int B\left(\theta \right)f_{c}\left(\theta \right)\;d\theta \;.$$

Note that the bias term B(θ) will typically be country-specific because the true IRF $P_{i}\left(\theta \right)=\Psi \left(\alpha_{i}\left(\theta \right)\right)$ are country-specific due to differential item functioning at the level of countries. Hence, item-specific relative country effects regarding the IRF that are uniformly weighted in Equation (15) can be considered a desirable property.

In the case of a fitted 2PL model, it holds that $a_{i}\left(\theta \right)=a_{i}\theta$, and deviations $\Psi \left(a_{i}\left(\theta \right)\right)-\Psi \left(\alpha_{i}\left(\theta \right)\right)$ are weighted by $a_{i}^{\prime }\left(\theta \right)=a_{i}$ in the derived bias in (15). For the 1PL model, the deviations are equally weighted due to $a_{i}^{\prime }\left(\theta \right)=1$. This property might legitimate the use of the often ill-fitting 1PL model because model deviations are equally weighted across items (see [27]). We elaborate on this discussion in the following Section 2.3.

### 2.3. A Few Remarks on the Choice of the IRT Model

In Section 2.1, we introduced several IRT models and it might be asked which criteria should be used for selecting one among these models. We think that model-choice principles depend on the purpose of the scaling models. Pure research purposes (e.g., understanding cognitive processes underlying item response behavior; modeling item complexity) must be distinguished from policy-relevant reporting practice (e.g., country rankings in educational LSA studies). Several researchers have argued that model choice should be primarily a matter of validity and not based on purely statistical criteria [27,60,61,62,63,64].

Myung et al. [63] discussed several criteria for model selection with a focus on cognition science. We would like to emphasize that these criteria might be differently weighted if applied to educational LSA studies that are not primarily conducted for research purposes. The concept of the interpretability of a selected IRT model means that the model parameters must be linked to psychological processes and constructs. We think that simple unidimensional IRT models in LSA studies are not used because one believes a unidimensional underlying (causal) variable exists. The chosen IRT model is used for summarizing item response patterns and for providing simple and interpretable descriptive statistics. In this sense, we have argued elsewhere [27] that model fit should not have any relevance for model selection in LSA studies. However, it seems in the official LSA publications such as those from PISA that information criteria are also used for justifying the use of scaling models [5]. We would like to note that these model comparisons are often biased in the sense that the personally preferred model is often the winner of this fit contest, and other plausible IRT models are excluded from these contests because they potentially could provide a better model fit. Information-criteria-based model selection falls into the criterion of generalizability according to Myung et al. [63]. These criteria are briefly discussed in Section 3.1.

Notably, different IRT models imply a differential weighting of items in the summary variable θ [29,65]. This characteristic is quantified with locally optimal weights (see Section 2.1). The differential item weighting might impair the comparison of subgroups. More critically, the weighing of items is, in most applications, determined by statistical models and might, hence, have undesirable consequences because practitioners have an implicitly defined different weighing of items in mind when composing a test based on a single test of items. Nevertheless, our study investigates the consequences of using different IRT models for LSA data. To sum up, which of the models should be chosen in operational practice is a difficult question that should not be (entirely) determined by statistical criteria.

## 3. Model Selection and Model Uncertainty

### 3.1. Model Selection

It is of particular interest to conduct model comparisons of the different scaling models that involve different IRFs (see Section 2.1). The Akaike information criterion (AIC) and the Bayesian information criterion (BIC) are used for conducting model comparisons in this article (see [66,67,68,69]). Moreover, the Gilula–Haberman penalty (GHP; [70,71,72]) is used as an effect size that is relatively independent of the sample size and the number of items. The GPH is defined as $\mathrm{GHP}=\mathrm{AIC}/(2\sum_{p=1}^{N}I_{p})$, where $I_{p}$ is the number of estimated model parameters for person *p*. The GHP can be seen as a normalized variant of the AIC. A difference in GHP larger than 0.001 is a notable difference regarding global model fit [72,73].

### 3.2. Model Uncertainty

Country comparisons in LSA studies such as PISA can depend on the chosen IRT model. In this case, choosing a single best-fitting model might be questionable [74,75]. To investigate the impact of model dependency, we discuss the framework of model uncertainty [76,77,78,79,80,81,82,83,84,85,86] in this section and quantify it by a statistic that characterizes model error.

To quantify model uncertainty, each model *m* is associated with a weight $w_{m}\geq 0$ and we assume $\sum_{m=1}^{M}w_{m}=1$ [87]. To adequately represent the diversity of findings from different models, an equal weighting of models has been criticized [88]. In contrast, particular models in the set of all models are downweighted if they are highly dependent and produce similar results [89,90,91]. We believe that model fit should not influence model weights [92]. The goal is to represent differences between models in the model error. If the model weights were determined by model fit, plausible but non-fitting models such as the 1PL model would receive a model weight of zero, which is not preferred because the 1PL model should not be excluded from the set of specified models. Moreover, if model weights are computed based on information criteria [80], only one or a few models receive weights that differ from zero, but all other models do not impact the statistical inference. This property is why we do not prefer Bayesian model averaging in our application [82,93,94].

Let $\gamma =(\gamma_{1},\ldots ,\gamma_{M})$ be the vector of a statistical parameter of all models. We can define a composite parameter $\gamma_{\mathrm{comp}}$ as

(17) $$\gamma_{\mathrm{comp}}=\sum_{m=1}^{M}w_{m}\gamma_{m}$$

We can also define a population-level model error (ME) as

(18) $$M_{\gamma_{\mathrm{comp}}}=\sqrt{\sum_{m=1}^{M}w_{m}{\left(\gamma_{m}-\gamma_{\mathrm{comp}}\right)}^{2}}$$

Now, assume that data is available and $\hat{\gamma }=\left({\hat{\gamma }}_{1},\ldots ,\gamma_{M}\right)$ is estimated. The estimate $\hat{\gamma }$ is multivariate normally distributed with mean γ and a covariance matrix V. Typically, estimates of different models using the same dataset will be (strongly) positively correlated. An estimate of the composite parameter $\gamma_{\mathrm{comp}}$ is given as

(19) $${\hat{\gamma }}_{\mathrm{comp}}=\sum_{m=1}^{M}w_{m}{\hat{\gamma }}_{m}$$

Due to $E\left({\hat{\gamma }}_{m}\right)=\gamma_{m}$, we obtain that ${\hat{\gamma }}_{\mathrm{comp}}$ is an unbiased estimate of $\gamma_{\mathrm{comp}}$. The empirical model error ME is defined as

(20) $$\mathrm{ME}=\sqrt{\sum_{m=1}^{M}w_{m}{\left({\hat{\gamma }}_{m}-{\hat{\gamma }}_{\mathrm{comp}}\right)}^{2}}$$

Now, it can be shown that ${\mathrm{ME}}^{2}$ is a positively biased estimate of $M_{\gamma_{\mathrm{comp}}}^{2}$ because the former also contains sampling variability. Define $\gamma_{\mathrm{comp}}=w^{⊤}\gamma$, where $w=(w_{1},\ldots ,w_{M})$. Similarly, we can write ${\hat{\gamma }}_{\mathrm{comp}}=w^{⊤}\hat{\gamma }$. Let $e_{m}$ be the *m*-th unit vector of length *M* that has an entry of 1 at the *m*-th entry and 0 otherwise. This notation enables the representation $\gamma_{m}=e_{m}^{⊤}\gamma$. Define $u_{m}=e_{m}-w$. From (18), we obtain

(21) $$M_{\gamma_{\mathrm{comp}}}^{2}=\sum_{m=1}^{M}w_{m}{\left(u_{m}^{⊤}\gamma \right)}^{2}=\sum_{m=1}^{M}w_{m}u_{m}^{⊤}\gamma \gamma^{⊤}u_{m}$$

Furthermore, we can then rewrite the expected value of $E({\mathrm{ME}}^{2})$ as (see Equation (20))

(22) $$E\left({\mathrm{ME}}^{2}\right)=M_{\gamma_{\mathrm{comp}}}^{2}+\sum_{m=1}^{M}w_{m}{\left(u_{m}\left(\hat{\gamma }-\gamma \right)\right)}^{2}=M_{\gamma_{\mathrm{comp}}}^{2}+\sum_{m=1}^{M}w_{m}u_{m}Vu_{m}^{⊤}=M_{\gamma_{\mathrm{comp}}}^{2}+B\;,$$

  where the second term B is a biasing term that is the estimated variation across models due to sampling error. This term can be estimated if an estimate of the covariance matrix V of the vector of model estimates $\hat{\gamma }$ is available. As an alternative, the bias in $E({\mathrm{ME}}^{2})$ can be removed by estimating B in (22) with resampling techniques such as bootstrap, jackknife or (balanced) half sampling [21,95]. Let $\hat{B}$ be an estimate of the bias; a bias-corrected model error can be estimated by

(23) $${\mathrm{ME}}_{\mathrm{bc}}=\sqrt{\max({\mathrm{ME}}^{2}-\hat{B},0)}$$

One can define a total error TE that includes the sampling error SE due to person sampling and a model error estimate ${\mathrm{ME}}_{\mathrm{bc}}$:

(24) $$\mathrm{TE}=\sqrt{{\mathrm{SE}}^{2}+{\mathrm{ME}}_{\mathrm{bc}}^{2}}$$

This total error also takes the variability in the model choice into account and allows for broader inference. Constructed confidence intervals relying on TE will be wider than ordinary confidence intervals that are only based on the SE.

## 4. Method

In our empirical application, we used data from PISA 2009 to assess the influence of the choice of different scaling models. Similar research with substantially fewer IRT modeling alternatives was conducted in [8,96,97].

### 4.1. Data

PISA 2009 data was used in this empirical application [3]. The impact of the choice of the scaling model was investigated for the three cognitive domains mathematics, reading, and science. In total, 35, 101, and 53 items were included in our analysis for the domains mathematics, reading, and science, respectively. All polytomous items were dichotomously recoded, with only the highest category being recoded as correct.

A total number of 26 countries were included in the analysis. The median sample sizes at the country level were Med = 5398 (M = 8578.0, Min = 3628, Max = 30,905) for reading, Med = 3761 (M = 5948.2, Min = 2510, Max = 21,379) for mathematics, and Med = 3746.5 (M = 5944.2, Min = 2501, Max = 21,344) for science.

For all analyses at the country level, student weights were taken into account. Within a country, student weights were normalized to a sum of 5000, so that all countries contributed equally to the analyses.

### 4.2. Analysis

We compared the fit of 11 different scaling models (see Section 2.1) in an international calibration sample [98]. To this end, 500 students were randomly sampled from each of the 26 countries and each of the three cognitive domains. Model comparisons were conducted based on the resulting samples involving 13,000 students.

In the next step, the item parameters obtained from the international calibration sample were fixed in the country-specific scaling models. In this step, plausible values for the θ distribution in each of the countries were drawn [99,100]. We did not include student covariates when drawing plausible values. Note that sampling weights were taken into account in this scaling step. The resulting plausible values were subsequently linearly transformed such that a weighted mean of 500 and a weighted standard deviation of 100 holds in the total sample of studies comprising all countries. Weighted descriptive statistics and their standard errors of the θ distribution were computed according to the Rubin rules of multiple imputation [3]. The only difference to the original PISA approach is that we apply balanced half sampling instead of balanced repeated replication for computing standard errors (see [21,101]). Balanced half sampling has the advantage of easy computation of the bias for model error (see Equation (23)).

For quantifying model uncertainty, model weights were assigned prior to analysis based on the principles discussed in  Section 3.2. First, because the 1PL, 2PL, and the 3PL are the most frequently used models in LSA studies, we decided that the sum of their model weight should at least exceed 0.50. Second, the weights of models with similar behavior (i.e., models that result in similar country means) should be decreased. These considerations resulted in the following weights: 1PL: 0.273, 2PL: 0.136, 3PL: 0.136; 1PCL: 0.061; 1PLL: 0.061; 1PGL: 0.061; 3PLQ: 0.068; 3PLRH: 0.068; 4PGL: 0.045; 4PL: 0.045; 4PLQ: 0.045. It is evident that a different choice of model weight will change the composite parameter of interest and the associated model error. We did not opt for a sensitivity analysis employing an alternative set of model weights in order to ease the presentation of results in this paper. In order to study the importance of sampling error (SE) and the bias-corrected model error (${\mathrm{ME}}_{\mathrm{bc}}$), we computed an error ratio (ER) that is defined by $\mathrm{ER}={\mathrm{ME}}_{\mathrm{bc}}/\mathrm{SE}$. Moreover, we computed the total error as $\mathrm{TE}=\sqrt{{\mathrm{SE}}^{2}+{\mathrm{ME}}_{\mathrm{bc}}^{2}}$.

All analyses were carried out with the statistical software R [102]. The different IRT models were fitted using the xxirt() function in the R package sirt [103]. Plausible value imputation was conducted using the R package TAM [104].

## 5. Results

### 5.1. Model Comparisons Based on Information Criteria

The 11 different scaling models were compared for the three cognitive domains mathematics, reading, and science for the PISA 2009 dataset. Table 1 displays model comparisons based on AIC, BIC, and ΔGHP, which is defined as the difference between the GHP values of a particular model and the best-fitting model.

Based on the AIC or ΔGHP, one of the models, 4PGL, 3PLQ, 3PLRH, 3PL, 4PL, or 4PLQ, was preferred in one of the domains. If the BIC were used as a selection criterion, the 3PLQ or the 3PLRH will always be chosen across the models. Notably, the operationally used 2PL model had only satisfactory for the reading domain. By inspecting ΔGHP, it is evident that the largest gain in model fit is obtained by switching from one- to two-, three- or four-parameter models. However, the gain in model fit from the 2PL to the 3PL model is not noteworthy.

In contrast, the gains in fitting the 3PLQ or 3PLRH can be significant. Among the one-parameter models, it is interesting that the loglog link function resulted in a better model fit for mathematics compared to the logistic or the cloglog link functions. This was not the case for reading or science. Overall, the model comparison for PISA 2009 demonstrated that the 3PLQ or 3PLRH should be preferred over the 2PL model for reasons of model fit.

### 5.2. Model Uncertainty for Distribution Parameters

**Figure 4:**
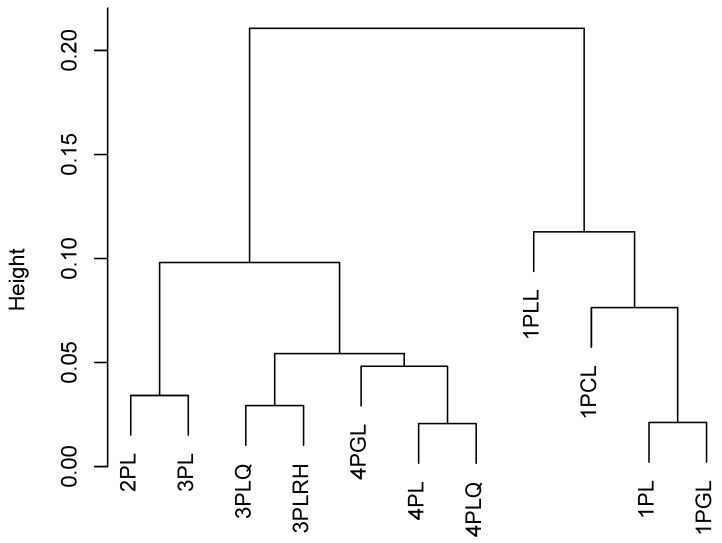
Dendrogram of cluster analysis using the Ward method for 11 different scaling models based on the distance matrix defined as average absolute differences between country means of models for PISA 2009 reading data.

To obtain a visual insight into the similarity of the different scaling models, we computed pairwise absolute differences in the country means. We used the average of them as a distance matrix used as the input of a hierarchical cluster analysis based on the Ward method. Figure 4 shows the dendrogram of this cluster analysis. It can be seen that the 2PL and 3PL provided similar results. Another cluster of models was formed by the more complex models 3PLQ, 3PLRH, 4PGL, 4PL, and 4PLQ. Finally, the different one-parameter models 1PLL, 1PGL, 1PL (and 1PGL) provided relatively distinct findings.

In Table 2, detailed results for 11 different scaling models for country means in PISA 2009 reading are shown The largest number of substantial deviations of country means from the weighted mean (i.e., the composite parameter) with at least 1 were obtained for the 1PCL model (10), 1PLL (9), and 4PLQ (9). At the level of countries, there were 11 countries in which none of the scaling models substantially differed from the weighted mean. In contrast, there was a large number of deviations for Denmark (DNK; 9) and South Korea (KOR; 10). The ranges in country means across different scaling models at the level of countries varied between 0.3 (SWE; Sweden) and 7.7 (JPN; Japan), with a mean of 2.4.

In Table A1 in Appendix C, detailed results for 11 different scaling models for country means in PISA 2009 mathematics are shown. The largest number of substantial deviations from the weighted mean was obtained for the 1PCL (12), the 1PLL (11), and the 1PGL (9) model. The ranges of the country means across models ranged between 0.5 and 7.9, with a mean of 2.8.

In Table A2 in Appendix C, detailed results for 11 different scaling models for country means in PISA 2009 science are shown. For science, many models showed a large number of deviations. This demonstrates large model uncertainty. The ranges of the country means across models varied between 0.6 and 7.8, with a mean of 2.8.

In Table 3, results and model uncertainty of 11 different scaling models for country means and standard deviations in PISA 2009 reading are shown. The unadjusted model error had an average of M = 0.66. The bias-corrected model error ${\mathrm{ME}}_{\mathrm{bc}}$ was slightly smaller, with M = 0.62. On average, the error ratio was 0.24, indicating that the larger portion of uncertainty is due to sampling error compared to model error.

The estimated country standard deviations for reading were much more model-dependent. The bias-corrected model error has an average of 0.96 (ranging between 0.00 and 2.68). This was also pronounced in the error ratio, which had an average of 0.60. The maximum error ratio was 2.05 for Finland (FIN; with a model error of 9.8), indicating that the model error was twice as large as the sampling error. Overall, model error turned out to be much more important for the standard deviation than the mean.

In Table 4, results and model uncertainty of 11 different scaling models for country 10th and 90th percentiles in PISA 2009 reading are shown. For the 10th percentile Q10, the error ratio was on average 0.60, with a range between 0.13 and 2.61. The average error ratio was even larger for the 90th percentile Q90 (M = 0.84, Min = 0.23, Max = 2.16). Hence, quantile comparisons across countries can be sensitive to the choice of the IRT scaling model.

In Table A3 in Appendix C, results and model uncertainty of 11 different scaling models for country means and standard deviations in PISA 2009 mathematics are shown. As for reading, the error ratio was on average smaller for country means (M = 0.24, Max = 0.66) than for country standard deviations (M = 0.77, Max = 1.58). Nevertheless, the additional uncertainty associated with model uncertainty is too large to be ignored in statistical inference. For example, South Korea (KOR) had a range of 15.7 for the standard deviation across models, which corresponds to an error of 3.75 and an error ratio of 1.58.

In Table A4 in Appendix C, results and model uncertainty of 11 different scaling models for country 10th and 90th percentiles in PISA 2009 mathematics are shown. The error ratios for the 10th and the 90th percentiles were similar (Q10: M = 0.66; Q90: M = 0.65). In general, the relative increase in uncertainty due to model error for percentiles was similar to the standard deviation.

In Table A5 in Appendix C, results and model uncertainty of 11 different scaling models for country means and standard deviations in PISA 2009 science are shown. As for reading and mathematics, the importance of model error was relatively small for country means (M = 0.27 for the error ratio). However, it reached 0.72 for Denmark with a bias-corrected model error of 1.89. For country standard deviations, the error ratio was larger (M = 0.53, Min = 0.00, Max = 1.50).

In Table A6 in Appendix C, results and model uncertainty of 11 different scaling models for country 10th and 90th percentiles in PISA 2009 science are shown. The influence of model error on percentiles was slightly smaller in science than in reading or mathematics. The average error ratios were M = 0.44 (Q10) and M = 0.57 (Q90), but the maximum error ratios of 1.53 (Q10) and 2.04 (Q90) indicated that model error was more important than sampling error for some countries.

To investigate the impact of the choice of model weights in our analysis (see Section 4.2), we additionally conducted a sensitivity analysis for the reading domain by using uniform model weights (weighting scheme W2). That is, we weighted each of the 11 scaling models by $w_{m}=1/11=0.091$ (m=1,…,11). We studied changes in country means and country standard deviations regarding the composite mean, standard errors (SE), and model errors (${\mathrm{ME}}_{\mathrm{bc}}$). The results are displayed in Table 5.

For the composite estimate of the country mean, we only observed tiny differences between the proposed model weighting W1 and the uniform weighting W2. The absolute difference in country means was 0.14 on average (SD = 0.11) and ranged between 0.01 and 0.36 (South Korea, KOR). The average absolute difference for the change in country standard deviations was also small (M = 0.26; SD = 0.20). Notably, there were almost no changes in the standard error for country means and country standard deviations for the weighting methods. However, the model error slightly increased with uniform weighting from M = 0.62 to M = 0.68 for country means and from 0.96 to 1.12 for country standard deviation. In conclusion, one can state that employing a different weighting scheme might not strongly change the composite estimate or the standard error but can have importance regarding the quantified model uncertainty in the model error ${\mathrm{ME}}_{\mathrm{bc}}$.

## 6. Discussion

Overall, our findings demonstrate that uncertainty regarding IRT scaling model influences country means. This kind of uncertainty is too large to be neglected in reporting. For some of the countries, the model error exceeded the sampling error. In this case, confidence intervals based on standard errors for the sampling of students might be overly narrow.

A different picture emerged for standard deviations and percentiles. In this case, the choice of the IRT model turned out to be much more important. Estimated error ratios were, on average, between 0.40 and 0.80, indicating that the model error introduced a non-negligible amount of uncertainty in parameters of interest. However, the importance of model error compared to sampling error was even larger for some of the countries. In particular, distribution parameters for high- and low-performing countries were substantially affected by the choice of the IRT model.

In our analysis, we only focused on 11 scaling models studied in the literature. However, semi- or nonparametric IRT models could alternatively be utilized [16,53,105,106,107], and their impact on distribution parameters could be an exciting topic for future research. If more parameters in an IRT model were included, we expect an even larger impact of model choice on distribution parameters.

In our analysis, we did not use student covariates for drawing plausible values [100,108]. It could be that the impact of the choice of the IRT model would be smaller if relevant student covariates were included [109]. Future research can provide answers to this important question. As a summary of our research (see also Section 2.3), we would like to argue that model uncertainty should also be reported in educational LSA studies. This could be particularly interesting because the 1PL, 2PL, or the 3PL models are applied in the studies. In model comparisons, we have shown that the 3PL with residual heterogeneity (3PLRH) and the 3PL with quadratic effects of θ (3PLQ) were superior to alternatives. If the 2PL model is preferred over the 1PL model for reasons of model fit, three-parameter models must be preferred for the same reason. However, a central question might be whether the 3PLRH should be implemented in the operational practice of LSA. Technically, it would be certainly feasible, and there is no practical added complexity compared to the 2PL or the 3PL model.

Interestingly, some specified IRT models have the same number of item parameters but a different ability to fit the item response data. For example, the 3PL and the 3PLRH models have the same number of parameters, but the 3PLRH is often preferred in terms of model fit. This underlines that the choice of the functional form is also relevant, not only the number of item parameters [30].

Frequently, the assumed IRT models will be grossly misspecified for educational LSA data. The misspecification could lie in the functional form of the IRFs or the assumption of invariant item parameters across countries. The reliance of ML estimation on misspecified IRT models might be questioned. As an alternative, (robust) limited-information (LI) estimation methods [110] can be used. Notably, ML and LI methods result in a different weighing of model errors [111]. If differential item functioning (DIF) across countries is critical, IRT models can also be separately estimated in each country, and the results brought onto a common international metric through linking methods [112,113]. In the case of a small sample size at the country level, regularization approaches for more complex IRT models can be employed to stabilize estimation [114,115]. Linking methods have the advantage of a clear definition of model loss regarding country DIF [116,117,118] compared to joint estimation with ML or LI estimation [119].

As pointed out by an anonymous reviewer, applied psychometric researchers seem to have a tendency to choose the best fitting model with little care for whether that choice is appropriate in the particular research context. We have argued elsewhere that the 1PL model compared to other IRT models with more parameters is more valid because of its equal weighting of items [27]. If Pandora’s box is opened via the argument of choosing a more complex IRT model due to improved model fit, we argue for a specification of different IRT models and an integrated assessment of model uncertainty, as has been proposed in this article. In this approach, however, the a priori choice of model weights has to be carefully conducted.

## Figures and Tables

**Table 1:** Model comparisons based on information criteria for the three ability domains—mathematics, reading and science—in PISA 2009.

	Mathematics			Reading			Science		
Model	AIC	BIC	ΔGHP	AIC	BIC	ΔGHP	AIC	BIC	ΔGHP
1PL	217510	217779	0.0059	413555	414317	0.0055	347819	348222	0.0062
1PCL	220022	220291	0.0122	414757	415519	0.0070	348756	349160	0.0077
1PLL	216882	217151	0.0043	416988	417751	0.0098	348984	349388	0.0081
1PGL	216784	217068	0.0041	413369	414146	0.0053	347804	348223	0.0062
2PL	215621	216144	0.0012	410032	**411541**	0.0011	344597	345389	0.0009
4PGL	**215142**	216188	**0.0000**	**409163**	412182	**0.0000**	**344064**	345648	**0.0000**
3PLQ	**215153**	**215938**	**0.0000**	409327	**411591**	**0.0002**	**344097**	**345285**	**0.0001**
3PLRH	**215174**	**215959**	**0.0001**	409275	**411539**	**0.0001**	**344083**	**345271**	**0.0000**
3PL	215486	216099	0.0009	409767	**411605**	0.0008	344420	**345362**	0.0006
4PL	**215179**	**216060**	**0.0001**	409296	411852	**0.0002**	**344105**	**345368**	**0.0001**
4PLQ	**215168**	216102	**0.0001**	**409245**	411913	**0.0001**	**344089**	345464	**0.0000**

Note. AIC = Akaike information criterion; BIC = Bayesian information criteria; DGHP = difference in Gilula–Haberman penalty (GHP) between a particular model and the best-fitting model in terms of GHP; For model descriptions see Section 2.1 and Equations (3) to (14). For AIC and BIC, the best-fitting model and models whose information criteria did not deviate from the minimum value by more than 100 are printed in bold. For DGHP, the model with the smallest value and models with DGHP values smaller than 0.0005 are printed in bold.

**Table 2:** Detailed results for all 11 different scaling models for country means in PISA 2009 reading.

CNT	M	rg	${\mathrm{ME}}_{\mathrm{bc}}$	1PL	1PCL	1PLL	1PGL	2PL	4PGL	3PLQ	3PLRH	3PL	4PL	4PLQ
AUS	515.2	1.25	0.29	515.1	515.8	514.8	515.2	515.7	515.2	515.2	515.5	515.0	515.0	514.5
AUT	470.8	2.36	0.65	470.2	**469.6**	470.6	470.1	470.9	**472.0**	471.6	471.7	470.6	471.6	**471.9**
BEL	509.5	2.91	0.78	508.9	**507.8**	509.4	508.8	509.7	**510.7**	510.4	510.5	509.4	**510.7**	**510.6**
CAN	525.0	1.79	0.43	525.1	525.6	525.2	525.1	525.4	524.3	524.5	524.8	524.9	524.0	**523.8**
CHE	501.7	1.27	0.39	501.3	501.3	501.0	501.4	501.5	502.3	502.3	502.2	501.8	502.3	502.3
CZE	479.9	0.89	0.27	479.5	480.2	479.5	479.6	480.1	480.0	480.0	479.8	480.4	480.1	480.0
DEU	498.5	1.83	0.39	498.2	499.3	**497.5**	498.5	498.4	499.0	498.9	498.9	498.7	498.8	499.1
DNK	493.7	5.46	1.58	**495.0**	**497.3**	492.9	**495.6**	**492.6**	**491.9**	**492.0**	**491.8**	493.5	**492.1**	**492.1**
ESP	480.1	1.43	0.43	480.0	480.7	479.5	480.1	480.3	479.6	479.8	479.6	480.9	479.7	479.7
EST	501.5	2.43	0.75	501.2	**502.8**	**500.4**	501.4	502.0	500.9	501.0	501.0	**502.8**	500.7	500.8
FIN	539.0	1.66	0.41	539.0	538.7	539.2	538.9	538.7	539.8	539.2	539.6	538.4	539.7	**540.1**
FRA	498.0	4.54	1.13	497.4	**495.1**	**499.0**	497.0	497.7	**499.4**	**499.4**	**499.5**	497.7	**499.6**	**499.3**
GBR	494.0	1.29	0.20	494.0	494.7	493.4	494.1	494.0	494.0	494.1	494.0	494.2	493.8	493.8
GRC	480.6	3.42	0.96	**481.7**	**479.6**	**482.8**	481.1	480.3	**479.4**	480.0	479.7	480.0	**479.6**	**479.6**
HUN	494.2	1.74	0.40	494.4	495.0	493.8	494.4	494.5	493.5	493.6	493.7	494.3	493.3	493.4
IRL	496.8	2.04	0.51	496.5	497.7	**495.7**	496.8	497.4	496.4	496.6	496.6	497.5	496.5	496.4
ISL	501.2	0.78	0.15	501.3	501.6	501.5	501.2	501.3	501.1	500.8	501.0	500.8	501.3	501.2
ITA	486.5	1.37	0.32	486.3	485.6	486.6	486.2	486.8	486.7	487.0	486.9	486.6	486.8	486.9
JPN	521.3	7.70	1.60	522.3	**517.7**	**525.4**	521.4	520.4	521.6	521.0	520.7	**519.8**	522.2	522.2
KOR	539.7	4.03	1.45	**541.3**	**541.4**	**541.5**	**541.2**	538.7	**538.2**	**538.5**	**538.7**	**538.5**	**537.4**	**537.6**
LUX	472.7	4.38	1.22	471.7	**470.0**	473.0	**471.3**	473.2	**474.4**	**474.2**	**474.4**	472.5	**474.0**	**474.2**
NLD	509.0	1.57	0.28	509.1	509.8	508.2	509.4	508.6	508.9	509.1	508.7	508.8	509.2	509.1
NOR	503.3	0.89	0.14	503.3	503.6	503.7	503.1	503.2	503.3	503.2	503.0	503.3	503.7	503.9
POL	501.7	2.24	0.72	501.0	501.2	**500.4**	501.3	502.2	502.0	502.5	502.2	502.7	502.2	502.1
PRT	489.2	2.79	0.70	489.4	**490.8**	**488.0**	489.8	489.3	488.3	488.5	488.4	489.9	488.3	488.3
SWE	497.0	0.34	0.00	496.9	497.0	497.0	496.9	496.9	497.2	497.0	497.1	496.9	497.1	497.2

Note. CNT = country label (see Appendix B); M = weighted mean across different scaling models; rg = range of estimates across models; ME_bc_ = bias-corrected estimate of model error based on balanced half sampling (see Equation (23)); For model descriptions see Section 2.1 and Equations (3) to (14). Country means that differ from the weighted mean of country means of the 11 different models more than 1 are printed in bold.

**Table 3:** Results and model uncertainty of 11 different scaling models for country means and country standard deviations in PISA 2009 reading.

		Country Mean							Country Standard Deviation						
CNT	*N*	M	rg	SE	ME	${\mathrm{ME}}_{\mathrm{bc}}$	ER	TE	M	rg	SE	ME	${\mathrm{ME}}_{\mathrm{bc}}$	ER	TE
AUS	14,247	515.2	1.2	2.51	0.32	0.29	0.12	2.52	104.7	2.6	1.45	0.68	0.64	0.44	1.59
AUT	6585	470.8	2.4	3.34	0.69	0.65	0.19	3.40	104.6	6.8	2.16	1.66	1.64	0.76	2.71
BEL	8500	509.5	2.9	2.49	0.80	0.78	0.32	2.61	107.5	3.1	1.92	0.69	0.65	0.34	2.02
CAN	23,200	525.0	1.8	1.49	0.45	0.43	0.29	1.55	95.6	4.6	1.12	1.18	1.18	1.05	1.62
CHE	11,801	501.7	1.3	2.72	0.42	0.39	0.14	2.75	99.7	0.8	1.67	0.23	0.00	0.00	1.67
CZE	6059	479.9	0.9	3.17	0.32	0.27	0.09	3.18	95.2	1.3	1.86	0.39	0.20	0.11	1.87
DEU	4975	498.5	1.8	3.05	0.42	0.39	0.13	3.08	100.1	1.3	2.01	0.30	0.00	0.00	2.01
DNK	5920	493.7	5.5	2.10	1.58	1.58	0.75	2.63	88.0	3.5	1.31	0.70	0.68	0.52	1.48
ESP	25,828	480.1	1.4	2.12	0.44	0.43	0.20	2.17	91.9	4.6	1.18	1.16	1.13	0.96	1.64
EST	4726	501.5	2.4	2.70	0.77	0.75	0.28	2.80	85.5	3.8	1.71	0.85	0.82	0.48	1.89
FIN	5807	539.0	1.7	2.27	0.43	0.41	0.18	2.30	91.5	9.8	1.31	2.68	2.68	2.05	2.98
FRA	4280	498.0	4.5	3.92	1.16	1.13	0.29	4.08	112.2	1.8	2.92	0.55	0.41	0.14	2.95
GBR	12,172	494.0	1.3	2.47	0.25	0.20	0.08	2.47	99.6	2.8	1.34	0.77	0.73	0.55	1.53
GRC	4966	480.6	3.4	4.26	1.01	0.96	0.23	4.37	99.8	5.4	2.09	1.46	1.38	0.66	2.50
HUN	4604	494.2	1.7	3.62	0.46	0.40	0.11	3.64	94.8	2.7	2.78	0.67	0.58	0.21	2.84
IRL	3931	496.8	2.0	3.24	0.55	0.51	0.16	3.28	98.8	4.2	2.63	1.24	1.19	0.45	2.89
ISL	3628	501.2	0.8	1.67	0.23	0.15	0.09	1.68	102.0	3.5	1.40	1.03	0.96	0.68	1.69
ITA	30,905	486.5	1.4	1.61	0.33	0.32	0.20	1.64	101.4	3.7	1.35	0.81	0.77	0.57	1.55
JPN	6082	521.3	7.7	3.71	1.62	1.60	0.43	4.04	107.3	8.0	3.16	1.59	1.52	0.48	3.50
KOR	4989	539.7	4.0	3.10	1.51	1.45	0.47	3.42	84.2	8.4	1.76	2.23	2.02	1.15	2.68
LUX	4622	472.7	4.4	1.19	1.23	1.22	1.02	1.70	109.3	8.0	1.21	2.01	1.99	1.65	2.33
NLD	4760	509.0	1.6	5.58	0.35	0.28	0.05	5.59	95.1	4.1	1.89	1.12	1.01	0.54	2.14
NOR	4660	503.3	0.9	2.61	0.22	0.14	0.06	2.61	96.8	3.7	1.55	0.98	0.93	0.60	1.81
POL	4917	501.7	2.2	2.72	0.72	0.72	0.26	2.81	92.8	3.6	1.32	0.90	0.84	0.63	1.56
PRT	6298	489.2	2.8	3.17	0.71	0.70	0.22	3.25	91.8	3.2	1.75	0.74	0.71	0.40	1.89
SWE	4565	497.0	0.3	3.00	0.09	0.00	0.00	3.00	103.6	1.7	1.63	0.42	0.27	0.17	1.66

Note. CNT = country label (see Appendix B); *N* = sample size; M = weighted mean across different scaling models; rg = range of estimates across models; SE = standard error (computed with balanced half sampling); ME = estimated model error (see Equation (20)); MEbc = bias-corrected estimate of model error based on balanced half sampling (see Equation (23)); ER = error ratio defined as ME_bc_/SE; TE = total error computed by $\mathrm{TE}=\sqrt{{\mathrm{SE}}^{2}+{\mathrm{ME}}_{\mathrm{bc}}^{2}}$ (see Equation (24)).

**Table 4:** Results and model uncertainty of 11 different scaling models for country 10th and 90th percentiles in PISA 2009 reading.

		Country 10th Percentile							Country 90th Percentile						
CNT	*N*	M	rg	SE	ME	${\mathrm{ME}}_{\mathrm{bc}}$	ER	TE	M	rg	SE	ME	${\mathrm{ME}}_{\mathrm{bc}}$	ER	TE
AUS	14,247	379.5	5.5	2.98	1.52	1.49	0.50	3.33	646.8	11.2	3.33	3.10	3.04	0.91	4.51
AUT	6585	332.9	20.5	4.82	5.37	5.32	1.10	7.18	602.8	4.8	3.64	1.26	1.07	0.30	3.79
BEL	8500	369.0	7.7	4.09	2.15	2.08	0.51	4.59	644.7	16.8	2.78	4.24	4.24	1.52	5.07
CAN	23,200	400.8	4.9	2.40	1.42	1.41	0.59	2.78	646.7	11.9	1.92	3.00	3.00	1.56	3.56
CHE	11,801	370.5	7.5	3.68	1.83	1.77	0.48	4.09	627.7	10.9	3.36	3.11	3.09	0.92	4.56
CZE	6059	357.5	8.4	4.67	2.19	2.13	0.46	5.14	603.3	6.2	3.18	1.58	1.53	0.48	3.53
DEU	4975	366.0	7.5	4.79	1.95	1.81	0.38	5.12	624.4	9.2	2.73	2.64	2.58	0.95	3.76
DNK	5920	378.2	4.1	2.82	0.96	0.91	0.32	2.96	604.0	4.7	2.57	1.45	1.43	0.56	2.94
ESP	25,828	359.0	8.7	3.24	2.18	2.12	0.66	3.87	595.1	3.0	1.86	0.78	0.74	0.40	2.00
EST	4726	390.9	7.3	3.83	1.81	1.76	0.46	4.21	610.7	6.2	3.17	1.50	1.46	0.46	3.49
FIN	5807	419.2	10.0	2.90	2.45	2.45	0.85	3.80	653.3	21.6	2.66	5.75	5.75	2.16	6.34
FRA	4280	350.5	13.8	5.93	3.68	3.59	0.60	6.93	638.6	16.3	4.92	3.88	3.82	0.78	6.23
GBR	12,172	365.9	9.9	3.00	2.57	2.57	0.86	3.95	621.7	5.0	3.01	1.45	1.39	0.46	3.31
GRC	4966	350.5	16.2	6.24	3.51	3.29	0.53	7.05	607.5	3.6	3.06	1.03	0.97	0.32	3.21
HUN	4604	368.6	7.0	6.08	1.56	1.40	0.23	6.24	613.4	4.5	4.08	1.21	1.12	0.28	4.23
IRL	3931	370.0	9.6	5.61	2.45	2.38	0.43	6.09	619.7	5.7	2.84	1.31	1.24	0.44	3.10
ISL	3628	366.3	6.0	2.67	1.40	1.28	0.48	2.96	628.2	11.2	2.33	2.84	2.76	1.18	3.62
ITA	30,905	352.4	12.2	2.65	2.67	2.65	1.00	3.75	613.7	7.7	1.86	2.01	2.00	1.07	2.73
JPN	6082	381.0	4.8	7.46	1.17	1.01	0.14	7.52	652.9	25.9	3.39	5.73	5.67	1.68	6.60
KOR	4989	430.5	13.8	4.18	3.53	3.31	0.79	5.33	644.5	14.7	3.51	3.68	3.60	1.02	5.03
LUX	4622	328.3	24.5	2.42	6.36	6.31	2.61	6.76	609.8	5.9	1.83	1.63	1.55	0.85	2.40
NLD	4760	386.8	3.5	5.84	0.91	0.73	0.13	5.89	632.7	12.9	5.35	3.47	3.36	0.63	6.31
NOR	4660	377.1	3.5	3.47	0.85	0.77	0.22	3.55	625.7	13.7	3.28	3.45	3.45	1.05	4.76
POL	4917	381.9	5.0	3.25	1.25	1.24	0.38	3.48	620.5	12.8	3.18	3.46	3.43	1.08	4.68
PRT	6298	369.9	6.6	4.51	1.43	1.34	0.30	4.70	606.8	3.5	3.20	0.83	0.74	0.23	3.29
SWE	4565	363.1	8.8	3.97	2.19	2.13	0.54	4.51	627.6	7.9	3.60	2.13	2.06	0.57	4.15

Note. CNT = country label (see Appendix B); *N* = sample size; M = weighted mean across different scaling models; rg = range of estimates across models; SE = standard error (computed with balanced half sampling); ME = estimated model error (see Equation (20)); MEbc = bias-corrected estimate of model error based on balanced half sampling (see Equation (23)); ER = error ratio defined as ME_bc_/SE; TE = total error computed by $\mathrm{TE}=\sqrt{{\mathrm{SE}}^{2}+{\mathrm{ME}}_{\mathrm{bc}}^{2}}$ (see Equation (24)).

**Table 5:** Sensitivity analysis for country means and country standard deviations for original and uniform model weighting for PISA 2009 reading.

	Country Mean						Country Standard Deviation					
	M		SE		${\mathrm{ME}}_{\mathrm{bc}}$		M		SE		${\mathrm{ME}}_{\mathrm{bc}}$	
CNT	W1	W2	W1	W2	W1	W2	W1	W2	W1	W2	W1	W2
AUS	515.2	515.2	2.51	2.51	0.29	0.33	104.7	104.7	1.45	1.46	0.64	0.74
AUT	470.8	471.0	3.34	3.33	0.65	0.74	104.6	104.3	2.16	2.18	1.64	1.90
BEL	509.5	509.7	2.49	2.49	0.78	0.90	107.5	107.6	1.92	1.91	0.65	0.74
CAN	525.0	524.8	1.49	1.49	0.43	0.53	95.6	95.8	1.12	1.13	1.18	1.34
CHE	501.7	501.8	2.72	2.73	0.39	0.43	99.7	99.7	1.67	1.68	0.00	0.00
CZE	479.9	479.9	3.17	3.16	0.27	0.20	95.2	95.2	1.86	1.86	0.20	0.15
DEU	498.5	498.7	3.05	3.04	0.39	0.44	100.1	100.1	2.01	2.00	0.00	0.03
DNK	493.7	493.4	2.10	2.10	1.58	1.75	88.0	87.8	1.31	1.33	0.68	0.84
ESP	480.1	480.0	2.12	2.11	0.43	0.44	91.9	91.5	1.18	1.16	1.13	1.34
EST	501.5	501.4	2.70	2.70	0.75	0.77	85.5	85.3	1.71	1.72	0.82	0.99
FIN	539.0	539.2	2.27	2.31	0.41	0.46	91.5	92.4	1.31	1.31	2.68	3.14
FRA	498.0	498.3	3.92	3.93	1.13	1.35	112.2	112.1	2.92	2.92	0.41	0.49
GBR	494.0	494.0	2.47	2.47	0.20	0.25	99.6	99.4	1.34	1.35	0.73	0.82
GRC	480.6	480.3	4.26	4.23	0.96	1.00	99.8	99.4	2.09	2.06	1.38	1.55
HUN	494.2	494.0	3.62	3.61	0.40	0.47	94.8	94.6	2.78	2.78	0.58	0.66
IRL	496.8	496.7	3.24	3.21	0.51	0.52	98.8	98.3	2.63	2.60	1.19	1.38
ISL	501.2	501.2	1.67	1.68	0.15	0.14	102.0	102.3	1.40	1.41	0.96	1.07
ITA	486.5	486.6	1.61	1.61	0.32	0.36	101.4	101.5	1.35	1.34	0.77	0.87
JPN	521.3	521.3	3.71	3.71	1.60	1.79	107.3	107.7	3.16	3.16	1.52	1.96
KOR	539.7	539.4	3.10	3.13	1.45	1.48	84.2	84.7	1.76	1.78	2.02	2.33
LUX	472.7	473.0	1.19	1.19	1.22	1.38	109.3	108.9	1.21	1.23	1.99	2.29
NLD	509.0	509.0	5.58	5.62	0.28	0.32	95.1	95.5	1.89	1.90	1.01	1.17
NOR	503.3	503.4	2.61	2.63	0.14	0.20	96.8	97.2	1.55	1.56	0.93	1.14
POL	501.7	501.8	2.72	2.73	0.72	0.67	92.8	93.0	1.32	1.34	0.84	0.96
PRT	489.2	489.0	3.17	3.16	0.70	0.83	91.8	91.5	1.75	1.74	0.71	0.88
SWE	497.0	497.0	3.00	3.00	0.00	0.00	103.6	103.4	1.63	1.64	0.27	0.32

Note. CNT = country label (see Appendix B); M = weighted mean across different scaling models; rg = range of estimates across models; SE = standard error (computed with balanced half sampling); ME_bc_ = bias-corrected estimate of model error based on balanced half sampling (see Equation (23)); W1 = model weighting used in the main analysis (see Section 4.2 and results in other tables); W2 = uniform weighting of models.

## Data Availability

The PISA 2009 dataset is available from https://www.oecd.org/pisa/data/pisa2009database-downloadabledata.htm (accessed on 13 March 2022).

## Abbreviations

    The following abbreviations are used in this manuscript:

AIC	Akaike information criterion
BIC	Bayesian information criterion
DIF	differential item functioning
GHP	Gilula-Haberman penalty
IRF	item response function
IRT	item response theory
LI	limited information
LSA	large-scale assessment
ME	model error
ML	maximum likelihood
PISA	programme for international student assessment
SE	standard error
TE	total error

## Appendix A. Ability Estimation in Misspecified IRT Models

Let $P_{i}\left(\theta \right)=\Psi \left(a_{i}\left(\theta \right)\right)$ be the true but unknown IRF, where Ψ is the logistic link function and $a_{i}$ is a differentiable function. If the IRFs are known, the latent ability θ can be obtained be maximizing the following log-likelihood function

(A1) $$l\left(\theta \right)=\sum_{i=1}^{I}\left\{x_{i}\log\Psi \left(a_{i}\left(\theta \right)\right)+\left(1-x_{i}\right)\log\left(1-\Psi (a_{i}\left(\theta \right))\right)\right\}\;.$$

The maximization of Equation (A1) provides the estimating equation

(A2) $$l_{1}\left(\theta_{0}\right)=\frac{\partial l}{\partial \theta }{|}_{\theta =\theta_{0}}=\sum_{i=1}^{I}\left(x_{i}-\Psi \left(a_{i}\left(\theta_{0}\right)\right)\right)a_{i}^{\prime }\left(\theta_{0}\right)=0\;,$$

where $a_{i}^{\prime }$ denotes the first derivative of $a_{i}$. Note that

(A3) $$E\left\{l_{1}\left(\theta_{0}\right)\right\}=\sum_{i=1}^{I}\left(\Psi \left(a_{i}\left(\theta_{0}\right)\right)-\Psi \left(a_{i}\left(\theta_{0}\right)\right)\right)a_{i}^{\prime }\left(\theta_{0}\right)=0\;,$$

Now assume that misspecified IRFs $P_{i}^{*}\left(\theta \right)=\Psi \left(\alpha_{i}\left(\theta \right)\right)$ instead of $P_{i}\left(\theta \right)$ are used. The following estimating equation provides an ability estimate $\theta_{1}$:

(A4) $$l_{1}^{*}\left(\theta_{1}\right)=\sum_{i=1}^{I}\left(x_{i}-\Psi \left(\alpha_{i}\left(\theta_{1}\right)\right)\right)\alpha_{i}^{\prime }\left(\theta_{1}\right)=0\;.$$

We make use of the following Taylor approximations

(A5) $$\alpha_{i}^{\prime }\left(\theta_{1}\right)≃\alpha_{i}^{\prime }\left(\theta_{0}\right)+\alpha_{i}^{\prime \prime }\left(\theta_{0}\right)\left(\theta_{1}-\theta_{0}\right)\;\mathrm{and}$$

(A6) $$\Psi \left(\alpha_{i}\left(\theta_{1}\right)\right)≃\Psi \left(\alpha_{i}\left(\theta_{0}\right)\right)+I\left(\alpha_{i}\left(\theta_{0}\right)\right)\alpha_{i}^{\prime }\left(\theta_{0}\right)\left(\theta_{1}-\theta_{0}\right)\;,$$

where I(x)=Ψ(x)(1−Ψ(x)). Set $\Delta \theta =\theta_{1}-\theta_{0}$. We obtain by inserting (A5) and (A6) in (A4)

(A7) $$l_{1}^{*}\left(\theta_{1}\right)≃\sum_{i=1}^{I}\left[x_{i}-\Psi \left(\alpha_{i}\left(\theta_{0}\right)\right)-I\left(\alpha_{i}\left(\theta_{0}\right)\right)\alpha_{i}^{\prime }\left(\theta_{0}\right)\Delta \theta \right]\left[\alpha_{i}\left(\theta_{0}\right)+\alpha_{i}^{\prime }\left(\theta_{0}\right)\Delta \theta \right]=0\;.$$

We can now determine the bias Δθ by solving $E(l_{1}^{*}\left(\theta_{1}\right))=0$ for $\theta_{1}$ and taking $E(l_{1}\left(\theta_{0}\right))=0$ into account. Moreover, we take the expectation and ignore the squared term in Δθ (i.e., Δθ≃0). Then, we compute from (A7)

(A8) $$\begin{array}{ll} & E\left\{l_{1}^{*}\left(\theta_{1}\right)\right\}\\ = & E\left\{\sum_{i=1}^{I}\left[x_{i}-\Psi \left(\alpha_{i}\left(\theta_{0}\right)\right)-I\left(\alpha_{i}\left(\theta_{0}\right)\right)\alpha_{i}^{\prime }\left(\theta_{0}\right)\Delta \theta \right]\left[\alpha_{i}^{\prime }\left(\theta_{0}\right)+\alpha_{i}^{\prime \prime }\left(\theta_{0}\right)\Delta \theta \right]\right\}\\ = & \sum_{i=1}^{I}\left[\Psi \left(a_{i}\left(\theta_{0}\right)\right)-\Psi \left(\alpha_{i}\left(\theta_{0}\right)\right)-I\left(\alpha_{i}\left(\theta_{0}\right)\right)\alpha_{i}^{\prime }\left(\theta_{0}\right)\Delta \theta \right]\left[\alpha_{i}^{\prime }\left(\theta_{0}\right)+\alpha_{i}^{\prime \prime }\left(\theta_{0}\right)\Delta \theta \right]\\ = & \sum_{i=1}^{I}\left[\Psi \left(a_{i}\left(\theta_{0}\right)\right)-\Psi \left(\alpha_{i}\left(\theta_{0}\right)\right)\right]\alpha_{i}^{\prime }\left(\theta_{0}\right)-\Delta \theta \sum_{i=1}^{I}\left\{I\left(\alpha_{i}\left(\theta_{0}\right)\right){\left[\alpha_{i}^{\prime }\left(\theta_{0}\right)\right]}^{2}-\left[\Psi \left(a_{i}\left(\theta_{0}\right)\right)-\Psi \left(\alpha_{i}\left(\theta_{0}\right)\right)\right]\alpha_{i}^{\prime \prime }\left(\theta_{0}\right)\right\}\\ = & 0 \end{array}$$

Finally, we obtain from (A8)

(A9) $$\theta_{1}=\theta_{0}+\frac{\sum_{i=1}^{I}\left[\Psi \left(a_{i}\left(\theta_{0}\right)\right)-\Psi \left(\alpha_{i}\left(\theta_{0}\right)\right)\right]\alpha_{i}^{\prime }\left(\theta_{0}\right)}{\sum_{i=1}^{I}\left\{I\left(\alpha_{i}\left(\theta_{0}\right)\right){\left[\alpha_{i}^{\prime }\left(\theta_{0}\right)\right]}^{2}-\left[\Psi \left(a_{i}\left(\theta_{0}\right)\right)-\Psi \left(\alpha_{i}\left(\theta_{0}\right)\right)\right]\alpha_{i}^{\prime \prime }\left(\theta_{0}\right)\right\}}\;.$$

We can further approximate the term in (A9) to

(A10) $$\theta_{1}≃\theta_{0}+A^{-1}\sum_{i=1}^{I}\left[\Psi \left(a_{i}\left(\theta_{0}\right)\right)-\Psi \left(\alpha_{i}\left(\theta_{0}\right)\right)\right]\alpha_{i}^{\prime }\left(\theta_{0}\right)$$

where $A=\sum_{i=1}^{I}I\left(\alpha_{i}\left(\theta_{0}\right)\right){\left[\alpha_{i}^{\prime }\left(\theta_{0}\right)\right]}^{2}$.

## Appendix B. Country Labels for PISA 2009 Study

The following country labels were used in the Results Section 5 for the PISA 2009 analysis:

AUS = Australia; AUT = Austria; BEL = Belgium; CAN = Canada; CHE = Switzerland; CZE = Czech Republic; DEU = Germany; DNK = Denmark; ESP = Spain; EST = Estonia; FIN = Finland; FRA = France; GBR = United Kingdom; GRC = Greece; HUN = Hungary; IRL = Ireland; ISL = Iceland; ITA = Italy; JPN = Japan; KOR = Republic of Korea; LUX = Luxembourg; NLD = Netherlands; NOR = Norway; POL = Poland; PRT = Portugal; SWE = Sweden.

## Appendix C. Additional Results for PISA 2009 Mathematics and Science

In Table A1, detailed results for 11 different scaling models for country means in PISA 2009 mathematics are shown. In Table A2, detailed results for 11 different scaling models for country means in PISA 2009 science are shown.

In Table A3, results and model uncertainty of 11 different scaling models for country means and standard deviations in PISA 2009 mathematics are shown. In Table A4, results and model uncertainty of 11 different scaling models for country 10th and 90th percentiles in PISA 2009 mathematics are shown. In Table A5, results and model uncertainty of 11 different scaling models for country means and standard deviations in PISA 2009 science are shown. In Table A6, results and model uncertainty of 11 different scaling models for country 10th and 90th percentiles in PISA 2009 science are shown.

**Table A1:** Detailed results for all 11 different scaling models for country means in PISA 2009 mathematics.

CNT	M	rg	${\mathrm{ME}}_{\mathrm{bc}}$	1PL	1PCL	1PLL	1PGL	2PL	4PGL	3PLQ	3PLRH	3PL	4PL	4PLQ
AUS	511.2	0.72	0.02	511.3	510.9	510.8	511.1	511.4	511.4	511.2	511.2	511.5	511.2	511.3
AUT	492.5	2.90	0.71	492.7	**491.2**	**494.1**	**493.9**	492.7	492.4	492.3	492.9	**491.2**	492.1	492.1
BEL	512.4	2.99	0.86	513.0	**511.3**	**514.2**	**514.2**	511.6	512.2	512.4	512.1	511.5	512.3	512.2
CAN	523.0	2.17	0.62	522.5	**521.9**	522.7	522.9	523.8	523.1	523.1	523.2	524.0	523.0	523.0
CHE	533.5	6.22	1.44	532.5	**529.0**	**535.0**	**535.2**	533.9	**534.5**	534.4	534.4	533.4	**534.9**	**534.6**
CZE	488.1	1.21	0.20	488.2	488.9	487.8	487.7	488.5	487.8	487.8	488.0	488.0	487.7	487.7
DEU	508.9	2.46	0.89	509.7	508.9	**510.4**	**510.3**	508.1	508.3	507.9	508.2	508.0	508.1	508.0
DNK	497.4	3.52	0.93	498.0	**499.7**	**496.3**	496.6	497.6	**496.2**	496.4	**496.2**	497.9	**496.4**	496.4
ESP	478.9	0.53	0.06	479.1	479.0	478.8	478.8	478.6	479.1	478.9	479.0	478.6	478.9	478.9
EST	508.1	5.35	1.35	507.6	**510.8**	**505.5**	**505.4**	508.9	507.8	507.9	507.7	**509.9**	507.9	507.9
FIN	538.1	5.13	1.27	**539.3**	**541.1**	538.2	537.9	537.9	**536.5**	**536.4**	**536.8**	538.2	**536.0**	**536.2**
FRA	490.8	1.79	0.50	491.3	490.0	491.6	491.8	490.0	490.4	490.7	490.6	490.4	490.5	490.5
GBR	486.9	2.30	0.53	486.6	486.9	**485.3**	**485.9**	487.1	487.1	487.3	487.1	487.6	487.3	487.3
GRC	458.0	3.95	0.97	458.6	457.6	**459.9**	**459.2**	457.3	458.3	458.0	458.2	**456.0**	457.9	457.8
HUN	483.4	1.11	0.00	483.5	484.1	483.1	483.0	483.5	483.5	483.2	483.4	483.1	483.3	483.4
IRL	482.6	1.97	0.55	482.1	482.1	**481.6**	482.0	483.1	483.0	483.0	482.7	483.6	483.2	483.2
ISL	501.0	3.02	0.74	501.5	**503.0**	500.1	500.2	500.7	500.0	500.4	500.1	501.3	500.3	500.4
ITA	478.0	0.88	0.18	478.1	478.6	478.1	477.8	477.7	478.2	478.2	478.2	477.8	478.2	478.2
JPN	529.9	3.06	1.11	**528.4**	529.1	529.1	528.9	530.5	**531.3**	**531.1**	**531.0**	530.5	**531.4**	**531.3**
KOR	544.7	7.87	2.45	**541.6**	**540.0**	**546.4**	**545.8**	545.6	**546.7**	**547.5**	**547.1**	545.6	**547.7**	**547.8**
LUX	483.4	1.55	0.46	483.8	482.8	484.1	484.0	482.7	483.7	483.3	483.7	482.5	483.4	483.5
NLD	521.5	1.98	0.51	522.0	**522.6**	521.4	521.5	521.2	520.8	520.8	520.8	521.5	520.6	520.7
NOR	493.3	4.11	0.87	493.4	**495.6**	**491.5**	**491.6**	493.5	492.9	493.0	492.8	493.9	493.0	493.0
POL	487.0	1.22	0.15	487.1	488.0	486.8	486.7	487.1	486.9	486.9	486.8	486.8	487.0	486.9
PRT	480.1	2.26	0.49	479.8	**478.7**	479.7	480.0	480.3	480.7	480.8	481.0	480.2	480.8	480.7
SWE	487.4	1.44	0.47	488.1	488.3	487.4	487.6	486.8	487.2	487.0	487.0	486.9	487.0	487.1

Note. CNT = country label (see Appendix B); M = weighted mean across different scaling models; rg = range of estimates across models; ME_bc_ = bias-corrected estimate of model error based on balanced half sampling (see Equation (23)); For model descriptions see Section 2.1 and Equations (3) to (14). Country means that differ from the weighted mean of country means of the 11 different models more than 1 are printed in bold.

**Table A2:** Detailed results for all 11 different scaling models for country means in PISA 2009 science.

CNT	M	rg	${\mathrm{ME}}_{\mathrm{bc}}$	1PL	1PCL	1PLL	1PGL	2PL	4PGL	3PLQ	3PLRH	3PL	4PL	4PLQ
AUS	517.6	2.73	0.83	518.4	518.1	**519.2**	518.3	516.7	517.3	517.1	517.2	**516.5**	517.2	517.1
AUT	488.1	1.11	0.18	487.9	488.6	487.6	488.0	488.4	488.3	488.4	488.7	487.9	488.3	488.2
BEL	498.1	2.37	0.55	497.8	**496.6**	498.9	497.7	498.5	498.6	498.5	498.5	498.2	498.7	498.6
CAN	519.6	0.65	0.09	519.6	519.5	520.0	519.6	519.4	519.6	519.6	519.5	519.6	519.4	519.6
CHE	509.2	0.96	0.35	508.7	508.8	508.9	508.7	509.5	509.6	509.7	509.7	509.4	509.6	509.6
CZE	494.1	2.89	0.98	**495.1**	**495.7**	494.5	**495.2**	493.5	**493.0**	**492.8**	**492.9**	493.6	**492.9**	**492.9**
DEU	513.9	2.13	0.53	514.2	**514.9**	514.7	514.2	514.0	513.3	513.1	513.5	513.7	513.1	**512.8**
DNK	488.3	4.70	1.89	**490.3**	**490.9**	**489.6**	**490.4**	**486.2**	**486.6**	**486.5**	**486.4**	**486.8**	**486.7**	**486.6**
ESP	478.2	2.07	0.42	478.2	479.0	**477.0**	478.4	478.1	477.8	477.9	477.7	478.7	478.0	478.0
EST	517.5	1.00	0.23	517.4	517.2	517.9	517.3	517.4	517.6	517.2	517.4	518.2	517.4	517.2
FIN	546.5	3.54	0.79	547.1	546.3	**549.0**	546.9	546.0	546.4	546.0	546.1	**545.5**	546.3	546.1
FRA	488.2	3.74	1.02	**487.2**	**485.9**	488.3	**487.1**	488.9	**489.3**	**489.6**	**489.5**	488.8	**489.3**	**489.5**
GBR	505.0	1.12	0.28	504.7	504.8	505.2	504.7	504.9	505.8	505.4	505.4	504.7	505.5	505.6
GRC	461.4	4.51	1.26	**460.3**	**458.3**	461.6	**460.0**	462.4	**462.8**	**462.5**	**462.5**	462.1	**462.5**	**462.5**
HUN	494.6	5.05	1.36	**495.8**	**498.0**	**493.5**	**496.1**	493.9	**492.9**	**493.0**	**493.0**	494.5	**493.0**	**493.1**
IRL	497.0	0.95	0.27	497.3	497.4	497.4	497.3	496.7	496.8	496.5	496.7	496.7	496.5	496.6
ISL	487.6	3.34	1.09	**486.5**	487.4	**485.5**	486.6	**488.8**	488.4	488.2	488.4	**488.8**	488.1	488.2
ITA	479.7	0.57	0.17	479.9	479.5	479.5	479.9	479.8	479.5	479.4	479.3	479.7	479.3	479.3
JPN	534.6	7.85	2.29	**532.4**	**530.2**	534.6	**532.1**	**536.1**	**536.3**	**537.6**	**536.9**	535.0	**538.1**	**537.6**
KOR	530.6	3.57	1.42	**529.1**	**529.0**	**529.1**	**529.2**	531.0	**532.0**	**532.5**	**532.4**	531.5	**532.3**	**532.4**
LUX	474.8	3.49	0.87	474.2	**472.6**	475.1	474.0	475.3	**476.1**	**476.0**	**476.1**	474.6	475.7	475.7
NLD	514.2	2.63	0.93	**515.2**	**515.6**	514.8	**515.2**	513.6	**513.1**	**513.0**	513.2	513.4	**513.0**	**513.1**
NOR	491.0	3.24	1.10	**492.2**	**492.6**	491.2	**492.3**	490.5	**489.4**	**489.6**	**489.4**	490.6	**489.6**	**489.7**
POL	499.6	3.08	0.70	500.0	**501.7**	498.6	500.2	499.3	499.0	498.9	499.0	499.7	498.7	498.9
PRT	483.4	4.41	0.88	483.2	**485.3**	**480.9**	483.5	483.8	483.0	483.1	482.9	484.4	483.1	483.1
SWE	487.3	1.54	0.34	487.1	**486.3**	487.2	487.0	487.5	487.6	487.9	487.7	487.5	487.9	487.9

Note. CNT = country label (see Appendix B); M = weighted mean across different scaling models; rg = range of estimates across models; ME_bc_ = bias-corrected estimate of model error based on balanced half sampling (see Equation (23)); For model descriptions see Section 2.1 and Equations (3) to (14). Country means that differ from the weighted mean of country means of the 11 different models more than 1 are printed in bold.

**Table A3:** Results and model uncertainty of 11 different scaling models for country means and country standard deviations in PISA 2009 mathematics.

		Country Mean							Country Standard Deviation						
CNT	*N*	M	rg	SE	ME	${\mathrm{ME}}_{\mathrm{bc}}$	ER	TE	M	rg	SE	ME	${\mathrm{ME}}_{\mathrm{bc}}$	ER	TE
AUS	9889	511.2	0.7	2.75	0.19	0.02	0.01	2.75	101.5	2.7	1.82	0.89	0.83	0.45	2.00
AUT	4575	492.5	2.9	3.17	0.80	0.71	0.22	3.25	105.1	6.0	2.05	1.76	1.68	0.82	2.65
BEL	5978	512.4	3.0	2.39	0.88	0.86	0.36	2.54	111.5	4.2	2.20	1.36	1.32	0.60	2.56
CAN	16,040	523.0	2.2	1.70	0.62	0.62	0.37	1.81	93.5	5.5	1.28	1.73	1.73	1.35	2.16
CHE	8157	533.5	6.2	3.59	1.45	1.44	0.40	3.87	105.2	7.2	1.85	2.33	2.29	1.23	2.94
CZE	4223	488.1	1.2	3.16	0.32	0.20	0.06	3.16	98.9	2.8	2.10	0.93	0.86	0.41	2.27
DEU	3503	508.9	2.5	3.45	0.91	0.89	0.26	3.56	104.6	2.3	2.27	0.86	0.73	0.32	2.38
DNK	4088	497.4	3.5	2.86	0.95	0.93	0.33	3.01	91.9	1.8	1.78	0.36	0.08	0.05	1.79
ESP	17,920	478.9	0.5	2.21	0.20	0.06	0.03	2.21	95.4	6.1	1.64	1.63	1.60	0.98	2.29
EST	3279	508.1	5.3	2.82	1.37	1.35	0.48	3.13	83.5	5.9	1.96	1.60	1.56	0.80	2.50
FIN	4019	538.1	5.1	2.22	1.32	1.27	0.57	2.56	87.8	8.4	1.82	2.61	2.59	1.42	3.17
FRA	2965	490.8	1.8	3.67	0.59	0.50	0.14	3.71	104.7	4.6	2.77	1.34	1.26	0.45	3.05
GBR	8431	486.9	2.3	2.77	0.59	0.53	0.19	2.82	94.2	3.1	1.75	0.90	0.82	0.47	1.93
GRC	3445	458.0	3.9	4.13	1.03	0.97	0.23	4.24	97.6	9.6	2.38	2.88	2.82	1.18	3.69
HUN	3177	483.4	1.1	4.04	0.26	0.00	0.00	4.04	97.8	5.4	3.42	1.69	1.69	0.49	3.82
IRL	2745	482.6	2.0	2.89	0.61	0.55	0.19	2.94	88.3	5.0	2.02	1.41	1.36	0.67	2.44
ISL	2510	501.0	3.0	2.14	0.76	0.74	0.35	2.26	95.0	2.5	2.09	0.69	0.61	0.29	2.18
ITA	21,379	478.0	0.9	2.09	0.24	0.18	0.09	2.10	98.0	5.5	1.40	1.32	1.32	0.94	1.92
JPN	4207	529.9	3.1	3.77	1.15	1.11	0.29	3.93	101.7	7.9	2.61	2.61	2.54	0.97	3.64
KOR	3447	544.7	7.9	3.71	2.52	2.45	0.66	4.45	94.0	15.7	2.38	3.90	3.75	1.58	4.45
LUX	3197	483.4	1.6	1.88	0.53	0.46	0.24	1.94	103.6	5.1	1.78	1.36	1.30	0.73	2.21
NLD	3318	521.5	2.0	5.19	0.56	0.51	0.10	5.22	96.4	4.5	2.06	1.57	1.49	0.73	2.54
NOR	3230	493.3	4.1	2.76	0.88	0.87	0.32	2.89	92.6	2.8	1.47	0.85	0.74	0.50	1.65
POL	3401	487.0	1.2	2.99	0.28	0.15	0.05	2.99	95.4	5.9	1.90	2.46	2.44	1.28	3.10
PRT	4391	480.1	2.3	2.99	0.54	0.49	0.16	3.03	97.7	4.7	1.93	1.53	1.49	0.77	2.44
SWE	3139	487.4	1.4	3.02	0.53	0.47	0.15	3.06	99.3	3.5	1.91	1.14	1.08	0.57	2.19

Note. CNT = country label (see Appendix B); *N* = sample size; M = weighted mean across different scaling models; rg = range of estimates across models; SE = standard error (computed with balanced half sampling); ME = estimated model error (see Equation (20)); MEbc = bias-corrected estimate of model error based on balanced half sampling (see Equation (23)); ER = error ratio defined as ME_bc_/SE; TE = total error computed by $\mathrm{TE}=\sqrt{{\mathrm{SE}}^{2}+{\mathrm{ME}}_{\mathrm{bc}}^{2}}$ (see Equation (24)).

**Table A4:** Results and model uncertainty of 11 different scaling models for country 10th and 90th percentiles in PISA 2009 mathematics.

		Country 10th Percentile							Country 90th Percentile						
CNT	*N*	M	rg	SE	ME	${\mathrm{ME}}_{\mathrm{bc}}$	ER	TE	M	rg	SE	ME	${\mathrm{ME}}_{\mathrm{bc}}$	ER	TE
AUS	9889	380.2	2.2	3.12	0.76	0.61	0.20	3.18	641.9	8.4	4.06	2.65	2.56	0.63	4.80
AUT	4575	355.5	16.2	4.22	4.74	4.60	1.09	6.25	627.1	7.3	3.86	2.24	2.09	0.54	4.39
BEL	5978	367.0	5.8	4.46	1.69	1.53	0.34	4.71	654.9	14.9	2.94	4.67	4.66	1.59	5.51
CAN	16,040	402.3	4.6	2.75	1.50	1.50	0.54	3.13	643.7	10.2	2.01	3.15	3.15	1.57	3.73
CHE	8157	393.9	3.6	4.29	1.14	0.97	0.23	4.40	666.6	20.3	4.16	5.76	5.71	1.37	7.07
CZE	4223	361.9	9.7	4.80	2.91	2.85	0.59	5.58	617.3	2.2	3.91	0.63	0.30	0.08	3.92
DEU	3503	371.8	6.7	5.12	1.89	1.85	0.36	5.45	642.6	11.6	3.75	3.83	3.75	1.00	5.30
DNK	4088	379.2	4.5	3.49	1.66	1.51	0.43	3.80	616.1	3.5	3.69	1.19	1.14	0.31	3.86
ESP	17,920	354.4	13.3	3.44	3.75	3.70	1.08	5.05	600.8	4.9	2.73	1.12	1.06	0.39	2.92
EST	3279	401.7	8.8	4.28	2.31	2.24	0.52	4.83	616.7	6.6	3.66	1.80	1.68	0.46	4.03
FIN	4019	425.0	8.7	3.39	2.66	2.61	0.77	4.28	650.9	13.8	3.12	4.60	4.57	1.47	5.54
FRA	2965	354.3	10.9	5.45	2.82	2.72	0.50	6.09	623.9	7.8	4.85	3.40	3.28	0.68	5.86
GBR	8431	366.8	6.3	3.32	2.16	2.09	0.63	3.92	609.5	2.0	3.93	0.58	0.26	0.07	3.94
GRC	3445	332.4	22.8	5.63	6.55	6.44	1.14	8.55	584.0	6.7	4.64	1.77	1.69	0.36	4.94
HUN	3177	356.7	12.3	6.07	3.57	3.57	0.59	7.04	608.5	6.4	6.03	1.63	1.51	0.25	6.21
IRL	2745	368.0	8.0	4.45	2.36	2.22	0.50	4.97	594.6	5.1	3.39	1.54	1.47	0.43	3.70
ISL	2510	378.3	3.4	3.70	1.25	1.05	0.28	3.84	622.2	4.7	3.46	1.70	1.60	0.46	3.82
ITA	21,379	351.5	11.3	2.47	3.41	3.41	1.38	4.21	604.3	4.4	2.89	0.91	0.83	0.29	3.01
JPN	4207	397.8	7.2	6.31	2.15	2.02	0.32	6.63	658.7	16.7	4.17	4.97	4.85	1.16	6.40
KOR	3447	424.9	10.6	4.52	3.22	2.92	0.65	5.38	666.7	32.2	5.09	8.04	7.88	1.55	9.38
LUX	3197	348.5	14.1	3.54	4.12	4.03	1.14	5.36	615.8	3.3	2.51	1.27	1.14	0.46	2.75
NLD	3318	396.9	4.6	5.92	1.18	0.92	0.16	6.00	645.8	10.1	5.02	3.31	3.22	0.64	5.96
NOR	3230	373.7	5.1	3.44	1.86	1.72	0.50	3.85	612.9	4.1	3.26	0.90	0.75	0.23	3.35
POL	3401	364.0	13.2	3.60	4.71	4.71	1.31	5.93	610.3	7.2	4.09	2.61	2.50	0.61	4.79
PRT	4391	354.5	12.0	3.47	3.68	3.64	1.05	5.02	607.0	2.6	4.22	0.74	0.49	0.12	4.25
SWE	3139	359.6	10.0	3.74	3.31	3.25	0.87	4.95	616.0	3.5	3.99	1.13	1.00	0.25	4.11

Note. CNT = country label (see Appendix B); *N* = sample size; M = weighted mean across different scaling models; rg = range of estimates across models; SE = standard error (computed with balanced half sampling); ME = estimated model error (see Equation (20)); MEbc = bias-corrected estimate of model error based on balanced half sampling (see Equation (23)); ER = error ratio defined as ME_bc_/SE; TE = total error computed by $\mathrm{TE}=\sqrt{{\mathrm{SE}}^{2}+{\mathrm{ME}}_{\mathrm{bc}}^{2}}$ (see Equation (24)).

**Table A5:** Results and model uncertainty of 11 different scaling models for country means and country standard deviations in PISA 2009 science.

		Country Mean							Country Standard Deviation						
CNT	*N*	M	rg	SE	ME	${\mathrm{ME}}_{\mathrm{bc}}$	ER	TE	M	rg	SE	ME	${\mathrm{ME}}_{\mathrm{bc}}$	ER	TE
AUS	9864	517.6	2.7	2.72	0.84	0.83	0.30	2.84	104.9	3.4	1.75	0.65	0.58	0.33	1.84
AUT	4577	488.1	1.1	3.64	0.29	0.18	0.05	3.64	105.7	2.2	2.91	0.63	0.53	0.18	2.96
BEL	5938	498.1	2.4	2.51	0.55	0.55	0.22	2.57	106.7	2.4	1.98	0.61	0.57	0.29	2.06
CAN	16,075	519.6	0.7	1.81	0.15	0.09	0.05	1.81	93.8	3.6	1.24	0.94	0.91	0.74	1.54
CHE	8215	509.2	1.0	3.01	0.40	0.35	0.12	3.03	98.9	2.1	1.82	0.48	0.35	0.19	1.86
CZE	4252	494.1	2.9	3.43	1.00	0.98	0.29	3.57	99.1	1.1	2.66	0.30	0.00	0.00	2.66
DEU	3477	513.9	2.1	3.08	0.55	0.53	0.17	3.12	103.3	5.3	2.25	1.09	1.05	0.47	2.48
DNK	4101	488.3	4.7	2.62	1.92	1.89	0.72	3.23	95.2	3.6	1.98	1.11	1.09	0.55	2.26
ESP	17,876	478.2	2.1	2.18	0.46	0.42	0.19	2.22	87.9	4.0	1.64	1.00	0.97	0.59	1.90
EST	3272	517.5	1.0	2.75	0.31	0.23	0.08	2.76	87.3	4.1	1.91	1.09	1.06	0.56	2.18
FIN	4016	546.5	3.5	2.48	0.84	0.79	0.32	2.61	92.8	10.9	1.55	2.35	2.33	1.50	2.80
FRA	2960	488.2	3.7	3.91	1.10	1.02	0.26	4.04	105.3	4.1	3.09	1.27	1.15	0.37	3.29
GBR	8413	505.0	1.1	2.78	0.36	0.28	0.10	2.79	102.6	1.9	1.85	0.64	0.58	0.31	1.94
GRC	3452	461.4	4.5	4.10	1.26	1.26	0.31	4.29	96.8	8.8	2.22	2.05	2.00	0.90	2.99
HUN	3193	494.6	5.0	3.46	1.43	1.36	0.39	3.72	89.8	2.5	2.92	0.59	0.50	0.17	2.97
IRL	2738	497.0	1.0	3.31	0.36	0.27	0.08	3.32	99.4	1.7	2.81	0.50	0.33	0.12	2.83
ISL	2501	487.6	3.3	2.01	1.09	1.09	0.54	2.28	99.5	5.1	1.89	1.17	1.13	0.60	2.20
ITA	21,344	479.7	0.6	1.82	0.21	0.17	0.09	1.83	99.1	5.9	1.49	1.20	1.20	0.81	1.91
JPN	4222	534.6	7.8	3.76	2.29	2.29	0.61	4.40	106.7	10.3	3.15	2.72	2.69	0.85	4.14
KOR	3451	530.6	3.6	3.30	1.42	1.42	0.43	3.59	86.9	7.9	1.93	2.41	2.34	1.21	3.04
LUX	3195	474.8	3.5	1.94	0.91	0.87	0.45	2.12	107.9	6.5	1.53	1.63	1.58	1.03	2.20
NLD	3323	514.2	2.6	5.77	0.98	0.93	0.16	5.85	99.7	4.7	2.32	1.20	1.11	0.48	2.57
NOR	3204	491.0	3.2	2.67	1.15	1.10	0.41	2.88	93.2	3.2	1.65	0.81	0.74	0.45	1.81
POL	3397	499.6	3.1	2.72	0.73	0.70	0.26	2.81	92.7	2.3	1.93	0.58	0.52	0.27	2.00
PRT	4336	483.4	4.4	3.06	0.89	0.88	0.29	3.19	86.0	4.2	1.54	0.89	0.85	0.55	1.76
SWE	3157	487.3	1.5	2.85	0.39	0.34	0.12	2.87	102.4	2.2	1.58	0.50	0.38	0.24	1.63

Note. CNT = country label (see Appendix B); *N* = sample size; M = weighted mean across different scaling models; rg = range of estimates across models; SE = standard error (computed with balanced half sampling); ME = estimated model error (see Equation (20)); MEbc = bias-corrected estimate of model error based on balanced half sampling (see Equation (23)); ER = error ratio defined as ME_bc_/SE; TE = total error computed by $\mathrm{TE}=\sqrt{{\mathrm{SE}}^{2}+{\mathrm{ME}}_{\mathrm{bc}}^{2}}$ (see Equation (24)).

**Table A6:** Results and model uncertainty of 11 different scaling models for country 10th and 90th percentiles in PISA 2009 science.

		Country 10th Percentile							Country 90th Percentile						
CNT	*N*	M	rg	SE	ME	${\mathrm{ME}}_{\mathrm{bc}}$	ER	TE	M	rg	SE	ME	${\mathrm{ME}}_{\mathrm{bc}}$	ER	TE
AUS	9864	383.3	3.8	3.19	1.09	1.01	0.32	3.34	650.3	12.7	4.07	2.85	2.76	0.68	4.92
AUT	4577	350.9	7.9	5.68	2.39	2.29	0.40	6.13	621.7	3.8	4.29	1.05	0.90	0.21	4.38
BEL	5938	358.7	7.1	4.18	1.96	1.96	0.47	4.62	632.9	11.5	2.89	2.28	2.25	0.78	3.66
CAN	16,075	398.5	1.4	2.59	0.51	0.36	0.14	2.62	638.7	10.2	2.25	2.43	2.39	1.06	3.29
CHE	8215	379.4	3.4	3.95	1.11	0.94	0.24	4.06	634.2	8.2	3.83	2.01	2.00	0.52	4.32
CZE	4252	366.8	6.1	5.49	1.49	1.36	0.25	5.66	621.4	4.0	4.26	1.03	0.93	0.22	4.36
DEU	3477	379.8	2.3	4.87	0.83	0.36	0.07	4.88	645.4	12.5	3.50	2.43	2.38	0.68	4.23
DNK	4101	366.8	6.5	3.64	1.98	1.92	0.53	4.12	610.9	6.4	3.59	2.48	2.45	0.68	4.34
ESP	17,876	365.2	7.1	3.45	1.68	1.65	0.48	3.82	590.3	3.8	2.49	0.98	0.90	0.36	2.65
EST	3272	404.3	3.1	4.05	1.06	0.99	0.24	4.16	629.1	9.5	3.32	2.06	2.02	0.61	3.89
FIN	4016	426.8	8.9	3.41	2.11	2.07	0.61	3.98	665.1	21.2	3.05	4.61	4.56	1.49	5.48
FRA	2960	349.7	14.1	6.26	3.65	3.43	0.55	7.14	619.2	6.0	4.70	1.39	1.26	0.27	4.87
GBR	8413	372.5	6.5	3.56	1.74	1.69	0.47	3.94	635.8	9.4	3.86	2.70	2.62	0.68	4.67
GRC	3452	336.7	20.4	6.02	4.52	4.43	0.74	7.47	584.8	5.3	4.01	1.43	1.31	0.33	4.22
HUN	3193	378.8	2.5	6.41	0.82	0.37	0.06	6.42	609.8	4.9	3.77	1.19	1.11	0.30	3.93
IRL	2738	370.3	7.3	5.60	2.08	1.93	0.34	5.93	623.2	4.3	4.01	1.09	1.01	0.25	4.13
ISL	2501	357.8	10.4	3.77	2.56	2.48	0.66	4.51	613.3	3.7	2.78	1.12	1.04	0.37	2.97
ITA	21,344	350.7	14.0	2.87	2.85	2.85	1.00	4.04	605.7	2.2	2.13	0.61	0.57	0.27	2.21
JPN	4222	390.5	5.6	7.55	1.48	1.26	0.17	7.66	663.4	27.8	3.40	6.97	6.94	2.04	7.72
KOR	3451	417.4	6.3	3.83	2.02	1.86	0.49	4.26	639.9	16.7	4.45	4.91	4.91	1.10	6.63
LUX	3195	334.6	18.6	2.98	4.62	4.55	1.53	5.44	612.2	1.6	2.67	0.49	0.18	0.07	2.68
NLD	3323	385.4	3.7	6.36	1.34	1.08	0.17	6.45	642.4	11.2	5.48	2.52	2.44	0.45	6.00
NOR	3204	371.1	5.8	3.31	1.42	1.32	0.40	3.56	611.3	3.5	3.58	1.23	1.07	0.30	3.74
POL	3397	380.6	3.7	3.81	0.93	0.86	0.22	3.90	619.7	3.6	3.51	1.06	0.93	0.26	3.63
PRT	4336	373.7	5.4	3.67	1.31	1.17	0.32	3.85	595.4	6.9	3.46	1.27	1.24	0.36	3.68
SWE	3157	355.5	10.4	3.42	2.27	2.20	0.64	4.07	617.5	7.3	3.62	1.81	1.75	0.48	4.02

Note. CNT = country label (see Appendix B); *N* = sample size; M = weighted mean across different scaling models; rg = range of estimates across models; SE = standard error (computed with balanced half sampling); ME = estimated model error (see Equation (20)); MEbc = bias-corrected estimate of model error based on balanced half sampling (see Equation (23)); ER = error ratio defined as ME_bc_/SE; TE = total error computed by $\mathrm{TE}=\sqrt{{\mathrm{SE}}^{2}+{\mathrm{ME}}_{\mathrm{bc}}^{2}}$ (see Equation (24)).
